# Identification of β-Secretase (BACE1) Substrates Using Quantitative Proteomics

**DOI:** 10.1371/journal.pone.0008477

**Published:** 2009-12-29

**Authors:** Matthew L. Hemming, Joshua E. Elias, Steven P. Gygi, Dennis J. Selkoe

**Affiliations:** 1 Center for Neurologic Diseases, Brigham and Women's Hospital and Harvard Medical School, Boston, Massachusetts, United States of America; 2 Department of Cell Biology, Harvard Medical School, Boston, Massachusetts, United States of America; Lehigh University, United States of America

## Abstract

β-site APP cleaving enzyme 1 (BACE1) is a transmembrane aspartyl protease with a lumenal active site that sheds the ectodomains of membrane proteins through juxtamembrane proteolysis. BACE1 has been studied principally for its role in Alzheimer's disease as the β-secretase responsible for generating the amyloid-β protein. Emerging evidence from mouse models has identified the importance of BACE1 in myelination and cognitive performance. However, the substrates that BACE1 processes to regulate these functions are unknown, and to date only a few β-secretase substrates have been identified through candidate-based studies. Using an unbiased approach to substrate identification, we performed quantitative proteomic analysis of two human epithelial cell lines stably expressing BACE1 and identified 68 putative β-secretase substrates, a number of which we validated in a cell culture system. The vast majority were of type I transmembrane topology, although one was type II and three were GPI-linked proteins. Intriguingly, a preponderance of these proteins are involved in contact-dependent intercellular communication or serve as receptors and have recognized roles in the nervous system and other organs. No consistent sequence motif predicting BACE1 cleavage was identified in substrates versus non-substrates. These findings expand our understanding of the proteins and cellular processes that BACE1 may regulate, and suggest possible mechanisms of toxicity arising from chronic BACE1 inhibition.

## Introduction

Alzheimer's disease is the most common neurodegenerative disorder, affecting more than 5 million Americans and over 30 million people worldwide. In the US alone, the disease accounts for an estimated $148 billion dollars annually in healthcare expenses [1]. Despite the growing understanding of the molecular processes that lead to this disease, there is as yet no disease-modifying treatment. Accumulation and deposition of the amyloid-β (Aβ) protein is thought to be a precipitating factor driving disease pathogenesis [2]. Aβ is known to be a toxic stimulus in a variety of model systems, and emerging experimental and clinical attempts to intervene in the disease process have shown preliminary success by preventing the production or enhancing clearance of the Aβ peptide [3], [4], [5], [6].

Aβ is produced from two proteolytic cleavages of the amyloid precursor protein (APP). The first of these is performed by β-secretase on the lumenal domain of APP, secreting the APP ectodomain (APPs) into the extracellular space. The second cleavage is executed by the intramembrane protease, γ-secretase, within the hydrophobic lipid bilayer. These sequential biochemical events are essential for Aβ formation, and thus these two proteases have become principal targets for pharmacological intervention in Alzheimer's disease.

β-site APP cleaving enzyme 1 (BACE1), or memapsin-2, is an aspartic protease of the pepsin family that was identified as the principal β-secretase responsible for Aβ generation nearly a decade ago [7], [8], [9], [10], [11], [12]. BACE1 is necessary for Aβ production *in vivo* [13], and genetic *BACE1* deficiency rescues amyloid pathology and deficits seen in APP transgenic mice [14]. Currently, BACE1 inhibitors are in development for the treatment of Alzheimer's disease [15]. Our understanding of the normal biological functions of BACE1 is far from complete, as the majority of efforts to study this protease have focused solely on its role in Aβ generation. BACE1 is thought to have loose substrate specificity, with preferences including a leucine residue at P1 and a polar residue at P1' positions [16], [17], [18]. However, the few known BACE1 substrates do not strictly adhere to cleavage recognition motifs emerging from these *in vitro* studies.

BACE1 is primarily expressed within the central nervous system and predominantly by neurons [8]. Because few substrates of BACE1 have been identified and *BACE1* knockout mice were initially described as having no phenotype, chronic inhibition of BACE1 has been proposed as a potentially attractive therapy for Alzheimer's disease. However, several recent studies have highlighted myelination, behavioral and synaptic abnormalities in *BACE1* knockout mice, suggesting that this protease plays important functions in the development and maintenance of the nervous system [19], [20], [21]. Which substrates BACE1 processes to regulate these complex phenotypes is unknown. These results highlight the limitations in our understanding of the normal functions of this protease and leave us unable to predict the adverse effects BACE1 inhibition may produce in humans.

In order to better understand the normal cellular functions of BACE1, we have utilized quantitative proteomic methods in a cell culture model to identify the range of proteins that are regulated by β-secretase processing. Using this approach, we have discovered a large number of novel proteins subject to β-secretase cleavage in two human epithelial cell lines. Interestingly, many of these proteins are involved in contact-dependent intercellular communication or serve as receptors and have recognized roles in the nervous system and other tissues. The vast majority of these proteins are of type I transmembrane topology, with one having type II topology and three having glycophosphatidylinositol (GPI) anchors. Our findings indicate that BACE1 has a strong preference for single-pass membrane bound proteins, but that the transmembrane and cytoplasmic domains do not play obligate roles in recognition by β-secretase. We validated a subset of the identified substrates and non-substrates in a cell culture model, either through analysis of the endogenous protein or by stably expressing the candidate. Analysis of the primary sequence of the BACE1 substrates suggests several potential sites of cleavage. However, as non-substrates also bear potential β-secretase cleavage sites, primary sequence appears not to be the principal determinant of substrate selection. Our results demonstrate the broad role of BACE1 activity in membrane protein turnover, and suggest that this protease may help regulate many diverse biological processes.

## Results

### Quantitative Proteomics of Conditioned Medium from Cells Expressing BACE1 Identifies Many Novel β-Secretase Substrates

Stable isotope labeling with amino acids in cell culture (SILAC), in which cells of different genetic backgrounds or experimental conditions are differentially grown in the presence of heavy or light amino acids, enables sensitive and quantitative comparisons between two proteomes using mass spectrometry. Previously, we used this experimental approach to identify novel substrates of the intramembrane aspartyl protease γ-secretase [22]. In the present study, we use similar proteomic methods, coupled with genetic overexpression of BACE1, to identify in an unbiased fashion the proteins regulated by β-secretase in two epithelial cell lines.

**Figure 1:**
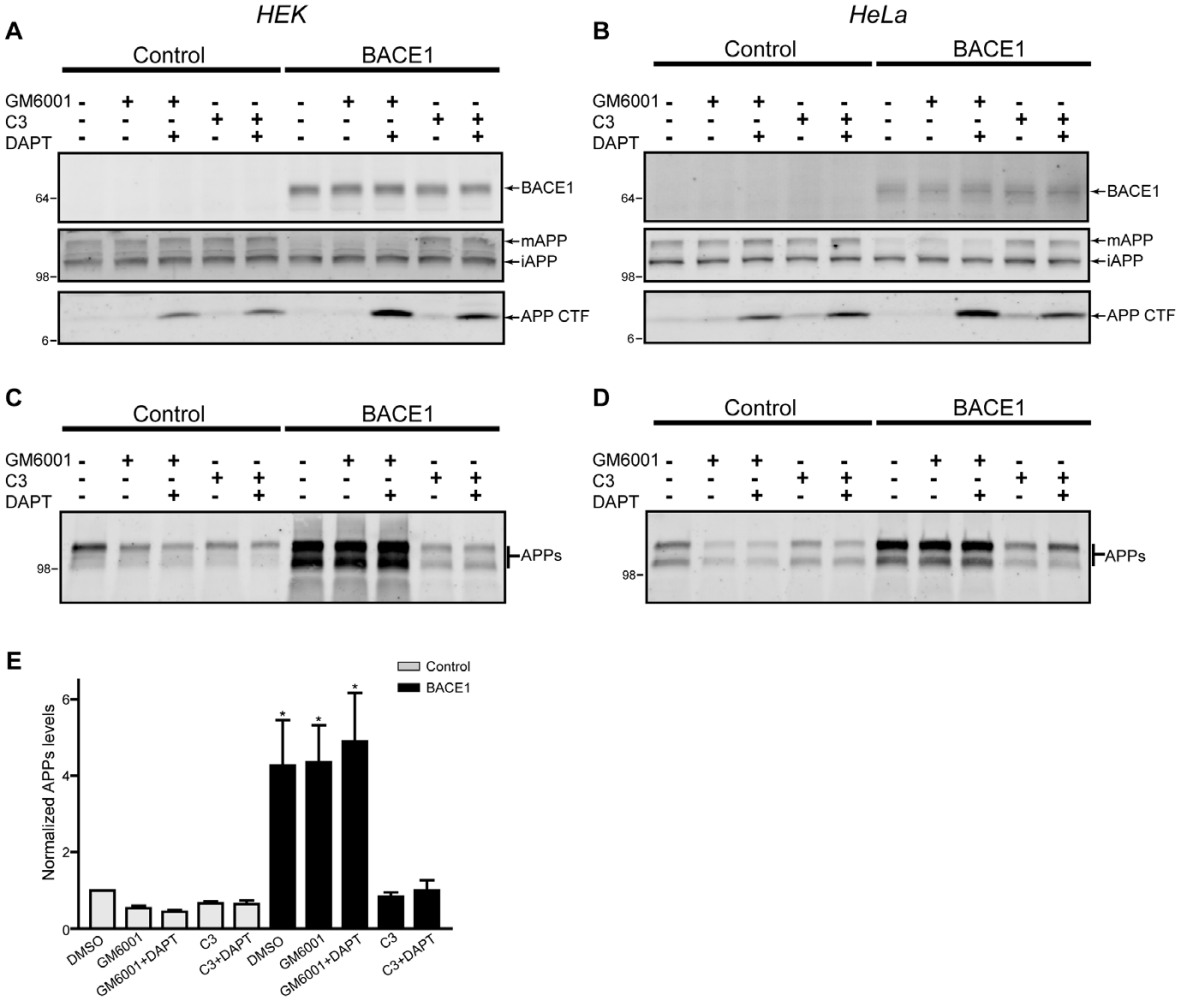
Characterization of cell lines expressing BACE1. HEK (left column) and HeLa (right column) cell lines were generated that stably express either BACE1 or an empty vector as control. To monitor changes in APP processing cells were treated with the metalloprotease (α-secretase) inhibitor GM6001, the β-secretase inhibitor C3 and the γ-secretase inhibitor DAPT. (A–B) Cell lysates were probed for the presence of myc-tagged BACE1 (top panel); endogenous full-length APP, which is present in a mature, fully glycosylated form (mAPP) and an incompletely glycosylated immature form (iAPP, middle panel); and membrane-bound APP CTFs (bottom panel) produced by ectodomain shedding. (C–D) Abundance of APPs in conditioned medium of the various treatment conditions. (E) Quantification of secreted APPs levels arising from the different treatments. Data from both cell lines were combined, and normalized to the control (DMSO) condition. The fold-accumulation of APPs arising from the various treatment conditions vs. DMSO control is graphed; * *p* < 0.05. “+” indicates the addition of a drug while “−” indicates the addition of DMSO as a control. The molecular weight in kDa is shown to the left of each Western blot panel.

The human cell lines used in this study were HEK and HeLa, derived from transformed embryonic kidney cells and cervical adenocarcinoma, respectively. HEK cells and, to a lesser extent HeLa cells, express a very low level of BACE1 protein, as evidenced by their modest capacity to produce Aβ. BACE1 is principally expressed within the nervous system [8], and endogenous BACE1 protein is undetectable by Western blot in these cell lines (see below). For these reasons, we chose to use a BACE1 overexpression system to enhance β-secretase activity and achieve levels of substrate shedding sufficient for proteomic identification. HEK and HeLa cell lines were each stably transfected with either myc tagged human BACE1 cDNA or an empty vector as control. HEK cells achieved higher BACE1 expression than HeLa cells (Figure 1A–B, top panels).

As an initial validation of this system for monitoring β-secretase activity, endogenous levels of the prototypical BACE1 substrate, APP, were analyzed (Figure 1). The metalloprotease (α-secretase) inhibitor GM6001, the β-secretase inhibitor C3 and the γ-secretase inhibitor DAPT were used to characterize the three principal proteolytic events in APP processing. Expression of BACE1 led to a clear decrease in the amount of mature APP in both HEK and HeLa cells, but the levels of immature, incompletely glycosylated APP remained unchanged (Figure 1A–B, middle panels). BACE1 shedding of mature APP was reversible upon application of a β-secretase inhibitor (C3). This finding indicates proper localization of β-secretase activity to post-Golgi compartments, where the enzyme typically resides on the cell surface and within the endosomal system [23]. APP C-terminal fragments (CTFs) are produced after ectodomain shedding by either α- or β-secretase enzymes. Normally, CTFs are rapidly turned over via intramembrane proteolysis by the γ-secretase complex, liberating an intracellular domain and a small secreted Aβ-like peptide. Upon γ-secretase inhibition with DAPT, CTFs accumulate to detectable levels, and CTF levels were further enhanced by the increased ectodomain shedding produced by BACE1 overexpression (Figure 1A–B, lower panels).

Using an ectodomain directed APP antibody, we analyzed the cellular conditioned medium for changes in secreted APP (APPs) arising from BACE1 expression (Figure 1C–D; quantification shown in Figure 1E). Under control conditions, APPs levels decrease by approximately 50% in response to metalloprotease (α-secretase) inhibition, and by approximately 30% in response to β-secretase inhibition. With BACE1 expression, APPs levels increase 4- to 5-fold above control, and this effect is entirely reversed by β-secretase inhibition with C3, confirming the specificity of our paradigm. Two distinct bands correspond to APPs, and both result from α- and β-secretase processing, as indicated by the inhibitor and overexpression experiments (Figure 1). Further, in the case of BACE1 expression, both bands arise from cleavage of the mature form of APP. Although the explanation for two distinct APPs species is unclear, they may arise from either different protein conformations that alter electrophoretic migration, or alternatively the occurrence of post-cleavage modification of APPs such as ectodomain phosphorylation [24], [25].

With these results validating the effects of BACE1 overexpression on one endogenous substrate, we next sought to probe the entire proteome of these two cell lines for proteins whose shedding was increased by elevated β-secretase activity. Stable BACE1 cells were metabolically labeled with heavy lysine and arginine, whereas control cells were labeled with the light form of these amino acids (see Materials and Methods for details). Once labeled and grown to confluence, cells were conditioned in serum-free medium containing 20 µM GM6001. This metalloprotease inhibitor was added to the conditioning medium of both BACE1 and control cells for two reasons. First, several of the known β-secretase substrates are subject to both metalloprotease (α-secretase) and β-secretase processing. As seen with APP (Figure 1E), addition of GM6001 enhances the difference in APPs levels between BACE1 and control conditions 2-fold. This enhanced difference in the abundance of BACE1-cleaved products was anticipated to increase our ability to detect potential substrates. Second, by decreasing basal metalloprotease shedding of proteins, particularly by the a disintegrin and metalloprotease (ADAM) and the matrix metalloprotease (MMP) family of enzymes, the complexity of the resultant conditioned medium may be reduced and thus improve our ability to detect low-abundance peptides.

Conditioned medium was collected from the BACE1 and control cells grown to equal confluence, and the medium combined in parallel for HEK and HeLa cells. This combined medium was then concentrated approximately 200-fold through a centrifugal filter device with a 3 kDa molecular weight cutoff. One hundred micrograms of protein from the concentrated conditioned medium was separated by SDS-PAGE, divided into ten horizontal slices, and subject to trypsinization and LC-MS/MS (see Figure S1 and Materials and Methods). Data arising from all quantitative peptide comparisons were analyzed to enrich for peptides demonstrating a relative abundance profile consistent with BACE1 expression. Proteins were considered as putative substrates when constituent peptides were found with at least 65% of the total (light plus heavy) signal derived from the BACE1 (heavy) condition. One hundred and sixteen proteins were identified that showed this degree of enrichment in the BACE1 conditioned medium, and these individual candidates were further evaluated.

Based on the known functions of BACE1 and the short list of established β-secretase substrates, we expected to find solely membrane-bound proteins enriched in the medium of BACE1 expressing cells. However, many of the proteins enriched in the BACE1 condition were not membrane-bound and are unlikely to be direct substrates of β-secretase. These are unlikely to be contaminants from lysed cells, as such an artifact should be present in equal abundance between the two conditions. An example of how such proteins may become enriched in the medium of BACE1 overexpressing cells is the co-secretion of a non-membrane-bound protein associated with another membrane protein that is itself cleaved by β-secretase. Alternatively, mistrafficking of a protein may occur after β-secretase cleavage of a receptor responsible for determining its localization. Unexpectedly, 43 of the 116 proteins elevated by BACE1 expression were soluble, non-membrane bound lysosomal proteins (Table S2). Investigation of the putative β-secretase substrates revealed the shedding of a membrane protein responsible for lysosomal trafficking (IGF2R/M6PR), indicating that these secreted lysosomal proteins were likely elevated in BACE1 conditioned medium due to the shedding of a receptor responsible for their trafficking (see below for further details). Peptides derived from BACE1 were found to be elevated in the media of cells expressing the protease, although we determined that BACE1 cannot shed itself (see below). Only four other non-membrane bound proteins were found to be elevated in the media of BACE1 expressing cells, and of these, three were related to collagen synthesis and one was a secreted protease inhibitor (Table S2).

**Figure 2:**
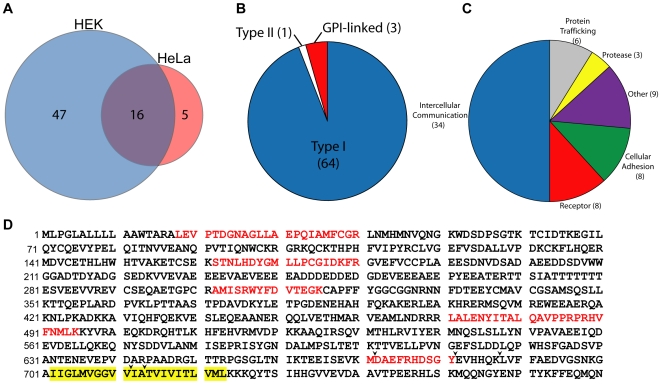
Regulation of single-pass and GPI-linked proteins by BACE1. Putative substrates identified to undergo BACE1 shedding in HEK and HeLa cells were examined for topology and proposed function. (A) Venn diagram indicating the total number of putative BACE1 substrates identified, and the number of these substrates that overlap or were unique to each cell type. (B) Membrane topology of the putative BACE1 substrates. (C) Putative BACE1 substrates were divided into several functional categories based on known protein functions and gene ontology classifications. (D) Sequence of APP, with peptides identified to be elevated by BACE1 expression indicated in red. The APP transmembrane sequence is highlighted in yellow, and arrowheads indicate the β- (major and minor sites), α-, γ-40 and γ-42 secretase cleavage sites (from left to right). APP-770 amino acid numbering is indicated on the left.

Of the 116 proteins elevated in the conditioned medium of BACE1 expressing cells, 68 were integral membrane proteins whose presence in the medium indicates their shedding (Table 1, additional information in Table S1). Analyzed by cell line, 47 putative substrates were found unique to HEK cells, five unique to HeLa cells, and 16 shared between the two cell lines (Figure 2A). All of these proteins were found to be either single-pass integral membrane proteins or GPI-linked proteins. The vast majority, 64 of 68, were type I transmembrane proteins. The four remaining putative substrates include three GPI-linked and one type II transmembrane protein (Figure 2B). Based on descriptions of protein function in the Gene Ontology and UniProt databases, these 68 substrates were divided into functional categories (Figure 2C). Many of the putative substrates are involved in contact-dependent intercellular communication by interacting with a membrane-bound cognate ligand on another cell. Members of this group include proteins implicated in neurodevelopment and migration, immune function, and cell fate determination. Other putative substrates have been described to function as peptide and lipoprotein receptors, cellular adhesion molecules, proteases, and in intracellular protein trafficking.

**Table 1:** Putative β-secretase substrates identified by quantitative proteomics.

Gene	Protein	Topology	Cell Line	PSMs	Unique PSMs	Ratio
APP	AMYLOID BETA A4 PROTEIN	Type I	HEK HeLa	169	8	0.89
APLP1	AMYLOID-LIKE PROTEIN 1	Type I	HEK HeLa	10	4	0.89
APLP2	AMYLOID-LIKE PROTEIN 2	Type I	HEK HeLa	165	9	0.96
IGF2R	CATION-INDEPENDENT MANNOSE-6-PHOSPHATE RECEPTOR	Type I	HEK HeLa	135	4	0.88
IL6ST	INTERLEUKIN-6 RECEPTOR BETA CHAIN	Type I	HEK HeLa	8	3	0.98
MET	HEPATOCYTE GROWTH FACTOR RECEPTOR	Type I	HEK HeLa	13	3	0.74
CPD	CARBOXYPEPTIDASE D	Type I	HEK	91	3	0.87
EPHA2	EPHRIN TYPE-A RECEPTOR 2	Type I	HEK	2	1	0.94
EPHA4	EPHRIN TYPE-A RECEPTOR 4	Type I	HEK	5	1	0.93
EPHA7	EPHRIN TYPE-A RECEPTOR 7	Type I	HEK	10	2	0.93
EPHB4	RECEPTOR PROTEIN TYROSINE KINASE VARIANT EPHB4V1	Type I	HEK HeLa	7	3	0.95
NCAM1	NEURONAL CELL ADHESION MOLECULE 1	Type I	HEK	11	4	0.88
L1CAM	NEURAL CELL ADHESION MOLECULE L1	Type I	HeLa	7	2	0.78
SEMA4B	SEMAPHORIN-4B	Type I	HEK HeLa	7	2	0.79
SEMA4C	SEMAPHORIN-4C	Type I	HEK	8	2	0.92
SEMA6A	SEMAPHORIN-6A	Type I	HEK	1	1	0.70
SEMA6D	SEMAPHORIN-6D	Type I	HEK	1	1	0.92
LRIG1	LEUCINE-RICH REPEATS AND IMMUNOGLOBULIN-LIKE DOMAINS PROTEIN 1	Type I	HEK	2	2	0.85
LRIG2	LEUCINE-RICH REPEATS AND IMMUNOGLOBULIN-LIKE DOMAINS PROTEIN 2	Type I	HEK	1	1	0.92
LRIG3	LEUCINE-RICH REPEATS AND IMMUNOGLOBULIN-LIKE DOMAINS PROTEIN 3	Type I	HEK	2	2	0.89
ROBO1	ROUNDABOUT HOMOLOG 1	Type I	HEK HeLa	27	3	0.81
ROBO2	ROUNDABOUT HOMOLOG 2	Type I	HEK	1	1	1.00
SDK1	SIDEKICK-1	Type I	HEK	14	1	0.88
SDK2	SIDEKICK-2	Type I	HeLa	8	2	0.91
PVR	POLIOVIRUS RECEPTOR	Type I	HEK HeLa	11	3	0.73
SORL1	SORTILIN-RELATED RECEPTOR	Type I	HEK	2	1	0.71
SORT1	SORTILIN	Type I	HEK	8	1	0.84
PCDH21	PROTOCADHERIN 21	Type I	HEK	10	2	0.76
PCDH7	PROTOCADHERIN 7	Type I	HEK	14	2	0.89
PCDHGA11	PROTOCADHERIN GAMMA A11	Type I	HEK	2	1	0.90
PCDHGA5	PROTOCADHERIN GAMMA A5	Type I	HEK	1	1	0.99
PCDHGA8	PROTOCADHERIN GAMMA A8	Type I	HEK	1	1	0.95
PCDHGC3	PROTOCADHERIN GAMMA C3	Type I	HEK	1	1	0.83
CD276	CD276 ANTIGEN	Type I	HEK	3	2	0.70
LRP11	LOW-DENSITY LIPOPROTEIN RECEPTOR-RELATED PROTEIN 11	Type I	HEK HeLa	9	3	0.91
LRP4	LOW-DENSITY LIPOPROTEIN RECEPTOR-RELATED PROTEIN 4	Type I	HEK	7	1	0.89
PAM	PEPTIDYL-GLYCINE ALPHA-AMIDATING MONOOXYGENASE	Type I	HEK	19	3	0.74
PODXL	PODOCALYXIN-LIKE PROTEIN 1	Type I	HEK	2	2	0.95
SDC4	SYNDECAN-4	Type I	HEK	10	4	0.96
BACE1	BETA-SECRETASE 1	Type I	HEK	10	4	0.90
HLA	HLA CLASS I HISTOCOMPATIBILITY ANTIGEN (Combined)	Type I	HEK HeLa	15	12	0.92
PLXDC2	PLEXIN DOMAIN-CONTAINING PROTEIN 2	Type I	HEK HeLa	10	3	0.81
BSG	BASIGIN	Type I	HEK HeLa	3	3	0.81
PTPRS	RECEPTOR-TYPE TYROSINE-PROTEIN PHOSPHATASE S	Type I	HEK	2	2	0.88
ALCAM	CD166 ANTIGEN	Type I	HEK HeLa	2	2	0.92
LMAN2	VESICULAR INTEGRAL-MEMBRANE PROTEIN VIP36	Type I	HEK	2	1	0.75
CACHD1	CACHE DOMAIN CONTAINING 1	Type I	HEK	45	1	0.84
SEZ6L2	SEIZURE 6-LIKE PROTEIN 2	Type I	HEK	19	1	0.92
NEO1	NEOGENIN	Type I	HEK	10	1	0.75
PRTG	PROTOGENIN	Type I	HEK	9	1	0.97
UNC5C	NETRIN RECEPTOR UNC5C	Type I	HEK	8	1	0.92
ITFG1	T-CELL IMMUNOMODULATORY PROTEIN	Type I	HEK	5	1	0.97
CSPG4	CHONDROITIN SULFATE PROTEOGLYCAN 4	Type I	HEK	4	1	0.68
TMEM132A	TRANSMEMBRANE PROTEIN 132A	Type I	HEK	3	1	0.72
ADAM10	DISINTEGRIN AND METALLOPROTEINASE DOMAIN-CONTAINING PROTEIN 10	Type I	HEK	2	1	0.68
BAMBI	BMP AND ACTIVIN MEMBRANE-BOUND INHIBITOR HOMOLOG	Type I	HEK	2	1	0.98
SPINT2	KUNITZ-TYPE PROTEASE INHIBITOR 2	Type I	HEK	2	1	0.96
DSG2	DESMOGLEIN 2	Type I	HEK	1	1	0.95
CRIM1	CYSTEINE-RICH MOTOR NEURON 1 PROTEIN	Type I	HEK	1	1	0.90
GLG1	GOLGI APPARATUS PROTEIN 1	Type I	HeLa	1	1	0.89
JAG1	JAGGED-1	Type I	HEK	1	1	0.97
LRRC33	LEUCINE-RICH REPEAT-CONTAINING PROTEIN 33	Type I	HeLa	1	1	1.00
TGOLN2	TRANS-GOLGI NETWORK INTEGRAL MEMBRANE PROTEIN 2	Type I	HEK	1	1	0.90
TLR9	TOLL-LIKE RECEPTOR 9	Type I	HeLa	1	1	0.66
TNFRSF21	TUMOR NECROSIS FACTOR RECEPTOR SUPERFAMILY MEMBER 21	Type I	HEK	1	1	1.00
CNTN1	CONTACTIN-1	GPI	HEK	4	2	0.87
EFNA5	EPHRIN-A5	GPI	HEK HeLa	2	2	0.71
GPC3	GLYPICAN-3	GPI	HEK	2	2	0.75
GOLIM4	GOLGI PHOSPHOPROTEIN 4	Type II	HEK	5	2	0.92

Columns indicate the gene and protein names, membrane topology, the cell line in which the protein was identified, the number of peptide spectral matches (PSMs) for the indicated protein, unique PSMs (indicating how many unique peptides were identified), and the average ratio of BACE1 peptides to total peptides identified. Data are sorted according to protein families.

As one would expect for a protein shed by β-secretase, all peptides that we identified should lie within the ectodomain of the protein, or possibly within an Aβ-like peptide if the remaining membrane-bound fragment undergoes intramembrane proteolysis. As an example of how these peptides map onto a protein, peptides derived from APP are illustrated in Figure 2D. As expected, all peptides lie within the ectodomain and are N-terminal to the known β-secretase cleavage sites. We mapped the majority of peptides identified by mass spectrometry to the full-length sequences of the putative β-secretase substrates, and as expected, all peptides correspond to the ectodomain region (Figure S2). The only exception was a peptide from the protein sidekick-2 that includes the entire transmembrane domain. Previously, twelve proteins have been implicated as β-secretase substrates [22], [26], [27], [28], [29], [30], [31], [32], and we were able to identify five of these (APP, APLP1, APLP2, LRP and DSG2) through our unbiased proteomic methods. The remaining 63 proteins we discovered are thus novel putative BACE1 substrates.

### Shedding of GPI-Linked and Type II Membrane Proteins by β-Secretase

To improve our confidence in the list of novel β-secretase substrates shown in Table 1, we sought to validate the BACE1-dependent shedding of several of the most interesting candidates. We used two methods in approaching the validation of these candidates: 1) probe with an antibody against the endogenous protein when available, or 2) clone, epitope tag and stably express the gene encoding the protein of interest. All validation experiments were performed in HEK cells that stably overexpress BACE1 or an empty vector as control.

Of the 68 proteins implicated as β-secretase substrates by proteomic identification, only four were non-type I transmembrane proteins. Three GPI-linked proteins, which undergo post-translational processing at the C-terminus that replaces a hydrophobic sequence with a glycophosphatidylinositol anchor, were identified as putative β-secretase substrates. To validate the β-secretase processing of a GPI-anchored protein, we examined ephrin-A5. Ephrins are cell-surface signaling molecules that bind in *trans* to Eph receptor tyrosine kinases to mediate intercellular communication. They have been studied largely in the context of neurodevelopment, in which they function to promote migration, attraction, repulsion and adhesion of cells and growth cones [33]. Ephrin-A5, in addition to signaling forward through its Eph cognate ligand, facilitates reverse signaling by forming compartmentalized microdomains [34]. Ephrins have previously been shown to undergo ADAM-dependent cleavage, which is thought to break the adhesive intercellular contact between Eph and ephrins to promote cellular detachment and repulsion [35].

**Figure 3:**
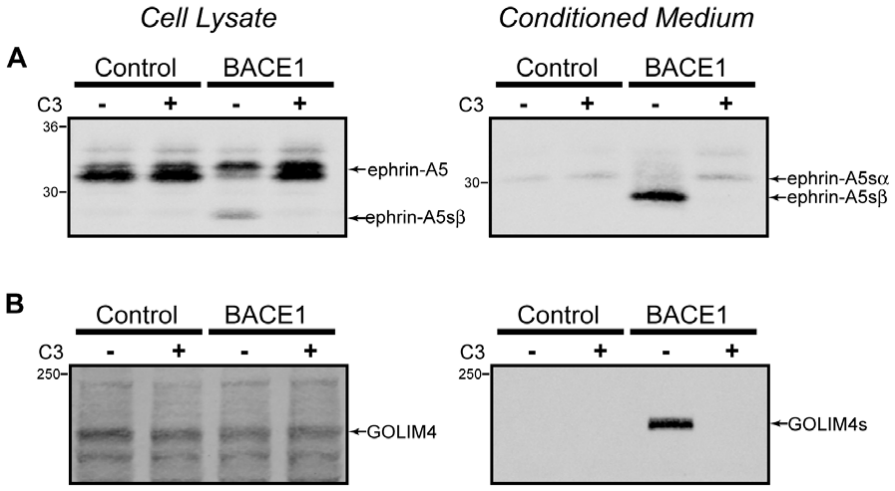
BACE1 shedding of GPI-linked and type II transmembrane proteins. Identified BACE1 substrates ephrin-A5 and GOLIM4 were cloned, FLAG-tagged, and stably expressed in HEK cells that express BACE1 or empty vector as control. The left column shows Western blots of cell lysates, and the right column shows blots of conditioned medium. Cells were treated with the β-secretase inhibitor C3 to confirm the necessity of BACE1 activity for ectodomain shedding. (A) Ephrin-A5, a GPI-linked protein, was robustly expressed and produced two prominent bands, the lower presumably representing the processed and mature GPI-linked form. BACE1 activity decreased the levels of full-length protein, and the shed product was visible within the cellular lysate (left panel). Conditioned medium revealed one minor (ephrin-A5sα) and one major (ephrin-A5sβ) band indicative of shed ephrin-A5, the major band corresponding to the BACE1 cleavage product (right panel). (B) GOLIM4, a type II transmembrane protein, was poorly expressed in cellular lysates (left panel), but accumulation of the shed ectodomain was found in conditioned medium of BACE1 expressing cells (right panel).

Ephrin-A5 was N-terminally FLAG tagged and stably expressed in HEK cells. Cellular lysates demonstrate robust expression of the full-length protein (Figure 3A, left panel). β-secretase activity decreased the level of the full-length protein, and produced a lower molecular weight band consistent with some shed ectodomain retained within the cell. Conditioned medium from ephrin-A5 expressing cells revealed the presence of a major shed band dependent on β-secretase activity (Figure 3A, right panel), establishing ephrin-A5 as a BACE1 substrate. In addition, a weak band of slightly higher molecular weight was observed and likely corresponds to the α-secretase (metalloprotease) shed ectodomain [35].

Golgi integral membrane protein 4 (GOLIM4), the sole type II transmembrane protein identified, has been shown to cycle between the Golgi apparatus and endosomes, where it functions in a bypass trafficking pathway that removes proteins from recycling endosomes to the Golgi [36]. In addition, GOLIM4 bypass trafficking is appropriated by Shiga toxin to facilitate toxin entry into the Golgi [37]. HEK cell lines stably expressing GOLIM4 produced only weak amounts of full-length protein, as shown by Western blotting (Figure 3B, left panel). The conditioned medium from these cells, however, showed robust accumulation of the shed GOLIM4 ectodomain that depended upon β-secretase activity (Figure 3B, right panel). These data demonstrate that BACE1 is capable of shedding some type II and GPI-linked proteins.

### Type I Transmembrane Proteins Are the Predominant Substrates of β-Secretase

As previous research identified BACE1 substrates only through candidate-based approaches, it has remained unclear whether the enzyme exhibits any preference for substrate topology. The large majority of the putative substrates we identified, numbering 64, were type I transmembrane proteins. These results suggest a strong intrinsic bias of β-secretase towards type I proteins, which may reveal some insight into how the enzyme recognizes its substrates. In addition to confirming the β-secretase processing of several type I transmembrane proteins identified as putative substrates, we also sought to scrutinize several candidate non-substrate proteins to observe if their abundance or processing changes in response to BACE1 expression.

**Figure 4:**
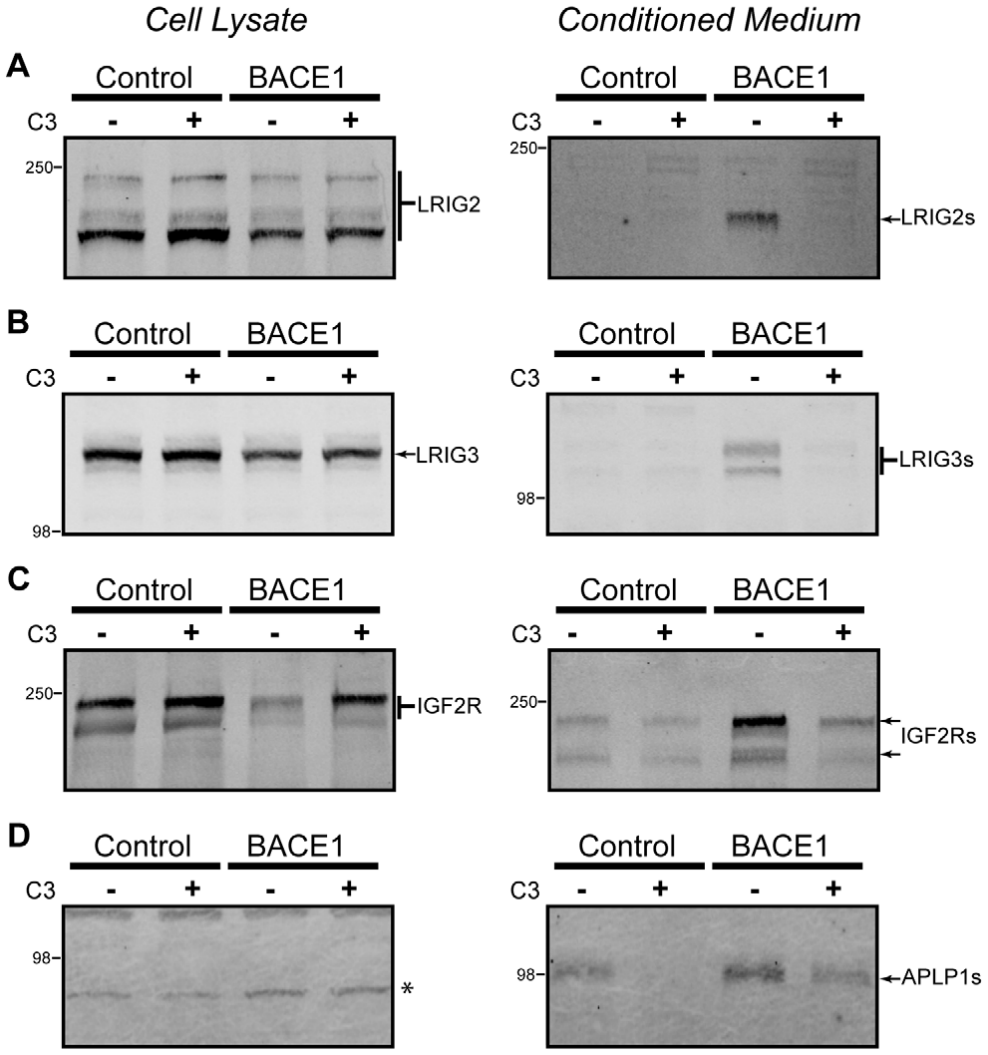
BACE1 shedding of type I transmembrane proteins. Identified BACE1 substrates of type I topology were either cloned and stably expressed or the endogenous protein was analyzed in HEK cells by Western blot. Cell lysates are shown in the left column, and conditioned medium shown in the right column. (A) LRIG2 was expressed in cell lysates as several distinct bands, likely owing to differential glycosylation (left panel). LRIG2 shedding by BACE1 was observed in the conditioned medium (right panel). (B) LRIG3 was stably expressed, as shown in cell lysates (left panel). LRIG3 was shed by BACE1 into the conditioned medium (right panel). (C) Endogenous IGF2R was analyzed with an ectodomain directed antibody. β-secretase activity produced a prominent decline in the full-length protein (left panel), and an increase in the shed ectodomain in the conditioned medium (right panel). (D) Endogenous APLP1 was expressed at undetectable levels in the cell lysate (left panel; the asterisk denotes a background band) but accumulated in the conditioned medium due to BACE1-mediated ectodomain shedding (right panel).

The leucine-rich repeats and immunoglobulin-like domains (LRIG) family is constituted by three type I integral membrane proteins that are broadly expressed in many tissues. The LRIG family has been found to antagonize growth factor signaling [38], and alterations in the expression of LRIG proteins is thought to play a role in tumorigenesis [39]. It has been demonstrated recently that a recombinant soluble ectodomain of LRIG1 antagonizes epidermal growth factor receptor signaling [40], although the physiological shedding of LRIG protein ectodomains has not previously been described. By mass spectrometry, we identified one unique peptide each of LRIG1 and LRIG3, and one peptide conserved in sequence between the three LRIG family members, each of which was elevated in conditioned media by β-secretase activity (Table 1). To validate that the LRIG family is indeed subject to β-secretase processing, we stably expressed N-terminally FLAG tagged LRIG2 and LRIG3 in HEK cells also stably expressing either BACE1 or an empty vector as control. Lysates of the LRIG2 and LRIG3 expressing cells modestly produced the full-length protein (Figure 4A–B, left panels), with LRIG2 being present in multiple bands likely arising from differential glycosylation. In the conditioned medium of LRIG2 and LRIG3 expressing cells, BACE1 expression produced an immunoreactive band indicative of ectodomain shedding (Figure 4A–B, right panels), and whose presence was sensitive to a β-secretase inhibitor. These results verify that BACE1 can cleave the LRIG family of proteins.

Insulin-like growth factor 2 receptor (IGF2R), also known as cation-independent mannose-6-phosphate receptor (M6PR), is a multifunctional glycoprotein. It serves as a receptor for IGF2, TGF-β, LIF, retinoic acid and other ligands [41]. Unlike the IGF1R, which signals upon ligand binding as a receptor tyrosine kinase, IGF2R has a smaller cytoplasmic domain with several potential phosphorylation sites but an incompletely characterized signaling function. The IGF2R has been proposed to function simply by binding and degrading ligands, but emerging evidence demonstrates that IGF2R is capable of signal transduction and that ligand binding modifies cellular behavior [42], [43]. Another well-defined role for this receptor is to bind newly synthesized soluble lysosomal proteins bearing mannose-6-phosphate, promote aggregation and internalization into clathrin-coated vesicles, and transport the bound lysosomal proteins to late endosomes for activation. The IGF2R protein is then recycled to the secretory pathway or the cell surface [44]. The existence of IGF2R shedding has been suggested by previous studies which have found soluble IGF2R in human serum, amniotic fluid and urine. By sequestering soluble growth factor ligands, the shed product may act as a negative regulator of growth [45].

We examined endogenously expressed IGF2R in HEK cells using an ectodomain directed antibody. In cell lysates, we detected the large IGF2R protein and found, like other BACE1 substrates, that the levels of full-length protein decrease with β-secretase activity (Figure 4C, left panel). In conditioned medium, shedding of the endogenously expressed IGF2R ectodomain was detectable under control conditions and was increased several fold with β-secretase activity (Figure 4C, right panel), thus validating IGF2R as a BACE1 substrate. The identification of IGF2R/M6PR as a substrate of β-secretase also provides a clear mechanistic explanation for the presence of soluble lysosomal enzymes in the conditioned medium of BACE1 expressing cells (see Discussion for further details).

We next examined β-secretase processing of endogenous amyloid precursor-like protein-1 (APLP1). Although APLP1 has already been identified as a BACE1 target [26], [46], its validation was of interest because APLP1 is expressed primarily in the central nervous system [47], and its shed ectodomain was unexpectedly identified here by mass spectrometry in the epithelial HEK and HeLa cell lines (Table 1). Using an ectodomain-specific antibody, we were unable to detect full-length APLP1 in cellular lysates (Figure 4D, left panel), consistent with low or nearly absent expression of this protein in epithelial cells. However, examination of concentrated conditioned medium revealed the presence of accumulated APLP1 ectodomain that depended on β-secretase activity (Figure 4D, right panel). These data highlight the sensitivity of the methods employed in this study, as we were able to detect BACE1-dependent processing of substrates expressed at undetectable levels in cellular lysates.

By mass spectrometry, we have identified a number of proteins involved in contact-dependent intercellular communication. Many of these newly identified β-secretase substrates may conceivably contribute to the observed *BACE1* knockout phenotype based upon their previously defined functions in the nervous system. We chose to examine the processing of semaphorins as a representative type I substrate involved in intercellular communication. The semaphorin family consists of eight classes of proteins that each have distinct domains and are either membrane bound or secreted. Different classes of semaphorins bind to unique receptors, which include plexins and neuropilins, and receptor binding induces signaling that regulates actin dynamics [48]. In addition to regulating neuronal migration and synaptic plasticity, semaphorins have also been found to be involved in the development of other organ systems [49]. We chose to examine the processing of semaphorin 4C (Sema4C), which is a type I transmembrane protein and has been found to interact with post-synaptic density proteins in the nervous system [50] and contribute to myoblast differentiation [51].

**Figure 5:**
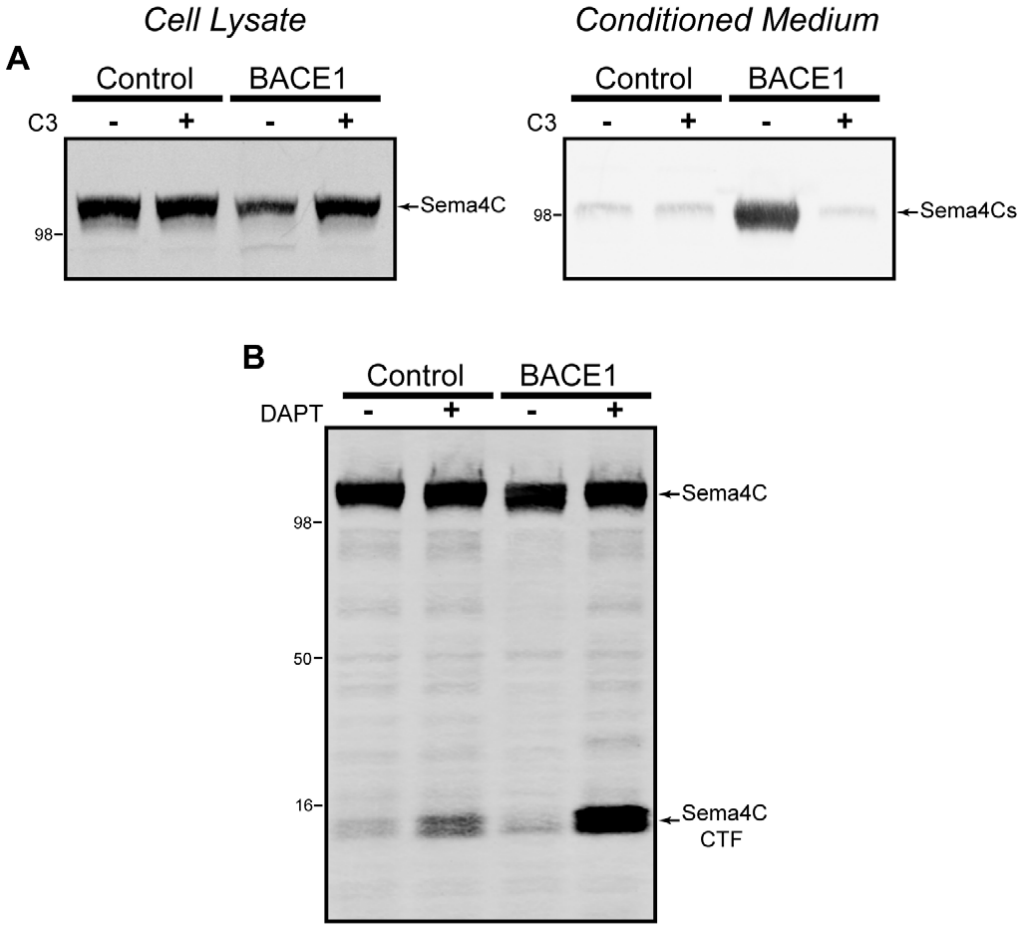
Semaphorin 4C is processed by BACE1 and γ-secretase. Semaphorin 4C was cloned with an N-terminal FLAG tag and a C-terminal HA tag, and stably expressed in HEK cells overexpressing BACE1 or empty vector as control. (A) Cell lysates show a single prominent band for mature Sema4C (left panel), which is shed by β-secretase activity into the medium (right panel). (B) Cells were treated with the γ-secretase inhibitor DAPT and cell lysates were probed for the presence of CTFs using the C-terminal HA epitope tag.

We stably expressed Sema4C in HEK cells also expressing either BACE1 or empty vector as a control. Probing for the N-terminal FLAG tag, we detected strong expression of Sema4C in cellular lysates, with levels of the full-length protein reduced by β-secretase activity (Figure 5A, left panel). Examination of the conditioned medium from these cells revealed a low level of shedding of Sema4C, which was greatly enhanced by increasing β-secretase activity (Figure 5A, right panel). In addition, we examined processing of the membrane-bound C-terminal fragment of Sema4C that is produced after ectodomain shedding by appending an HA epitope tag to the C-terminus. Anticipating that, like APP and many other type I transmembrane proteins, the Sema4C CTF would be processed by γ-secretase, we applied the γ-secretase inhibitor DAPT. In control cells, and to a greater extent in BACE1 expressing cells, we found Sema4C CTF levels to dramatically increase with DAPT inhibitor treatment, indicating the processing of Sema4C by γ-secretase (Figure 5B).

**Figure 6:**
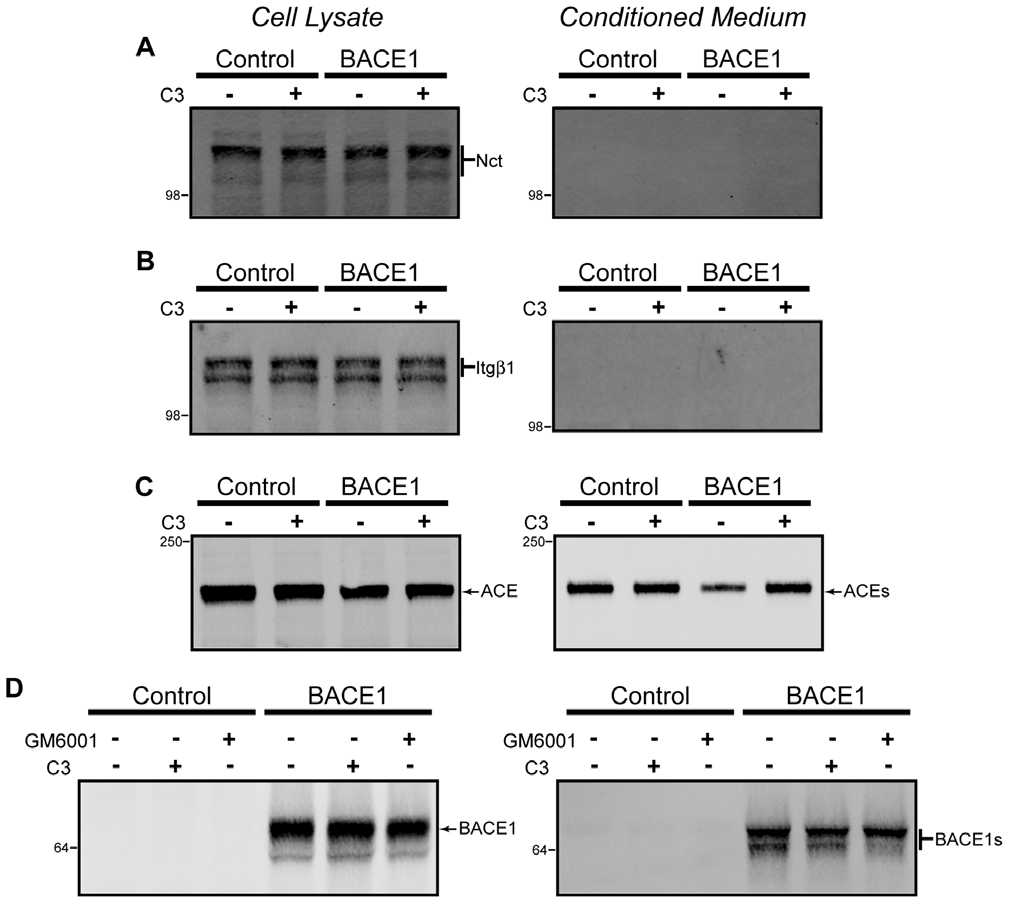
Single pass membrane proteins unaltered by β-secretase activity. Endogenous membrane proteins nicastrin (Nct, A) and Itgβ1 (B) were found not to be shed by BACE1 or another secretase. ACE (C) and BACE1 (D) were both shed by metalloproteases, but not affected by β-secretase activity. Left panels are from cell lysates, and right panels are from conditioned medium.

In addition to validating putative substrates, we also examined several candidate non-substrates for changes in protein abundance or aberrant shedding arising from BACE1 expression. These proteins were not enriched in conditioned medium by BACE1 expression as determined by mass spectrometry and have previously not been found to undergo β-secretase processing. We examined endogenous levels of two well studied type I transmembrane proteins not known to be shed: nicastrin (Nct) and integrin β-1 (Itgβ1). In both cases, BACE1 expression did not change the levels of protein in the cellular lysates and did not produce ectodomain shedding into the conditioned medium (Figure 6A–B). Additionally, we used the same stable expression model as was used for validating substrates to examine angiotensin-converting enzyme (ACE), which is shed by metalloproteases but not known to be shed by β-secretase [52], [53]. Lysates from cells overexpressing ACE showed comparable abundance of the full-length protein (Figure 6C, left panel). Shed ACE was found in the conditioned medium, as expected, although levels were found to be *decreased* by β-secretase activity (Figure 6C, right panel). The reduction in ACE shedding is likely the consequence of β-secretase cleavage of a metalloprotease contributing to ACE cleavage (e.g. ADAM10 [Table 1] or a similar metalloprotease). These results indicate that ACE is not a BACE1 substrate, but that BACE1 may indirectly regulate the shedding of non-substrates through the β-secretase processing of other ectodomain proteases.

By mass spectrometry, we found four unique peptides that correspond to the BACE1 ectodomain that were elevated with BACE1 expression (Table 1). Previous reports have identified BACE1 shedding, with a metalloprotease being the responsible enzyme [54]. To replicate this finding and establish whether BACE1 could shed itself, we used an ectodomain directed antibody to probe for soluble BACE1. In cellular lysates, we observed robust BACE1 expression upon transfection, but endogenous BACE1 was undetectable in HEK cells (Figure 6D, left panel). In the conditioned medium, one major and one minor immunoreactive band was detected. The minor band was insensitive to β-secretase inhibition, but did decrease with GM6001, a broad spectrum metalloprotease (α-secretase) inhibitor (Figure 6D, right panel). The major band, however, was insensitive to both metalloprotease and β-secretase inhibition, suggesting that an additional protease contributes to BACE1 shedding, and that BACE1 is unable to shed itself.

**Figure 7:**
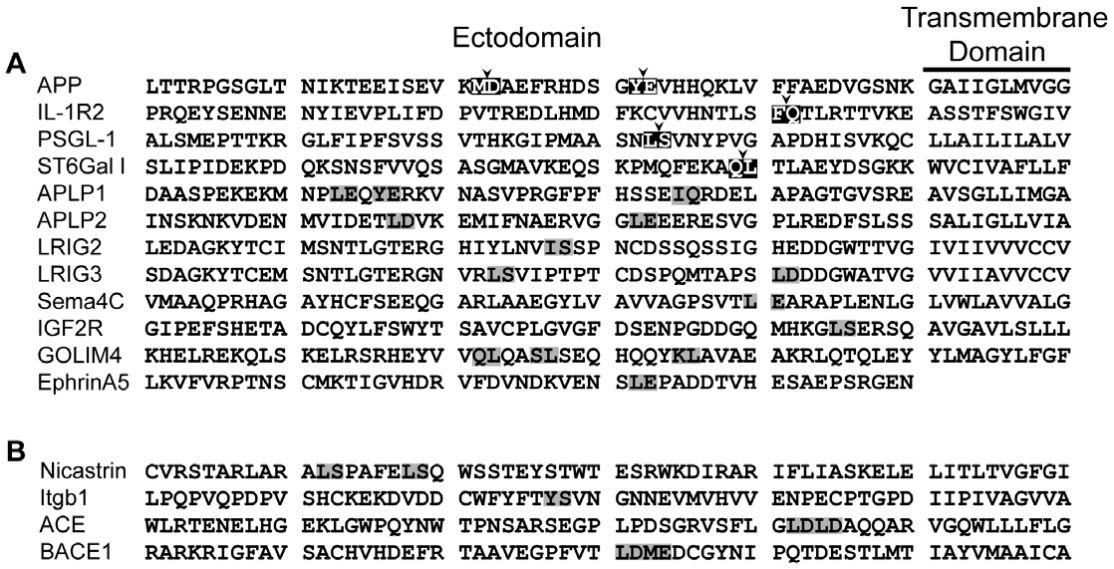
Alignment of β-secretase substrates and putative cleavage sites. The primary amino acid sequence of BACE1 substrates (A) and non-substrates (B) are shown. The first ten amino acids of the transmembrane domain are included, if present. Known cleavage sites are indicated with a black box and arrowhead, and potential cleavage sites are in gray. All sequences are human, except ST6Gal I, whose cleavage site was determined in rat. Sequence from type II proteins is listed from C- to N-terminal to maintain membrane orientation.

These results demonstrate that BACE1 is responsible for shedding numerous substrates, but that not all type I membrane proteins, even if shed by other secretases, can undergo β-secretase processing. Though a few BACE1 substrate cleavage sites have been mapped [27], [28], [29] and other studies have examined BACE1 cleavage of peptide libraries (see Discussion for details), little predictive information is available to aid in identifying putative substrates *a priori*. Our analysis of the sequences of several substrates reveals that all contain potential BACE1 cleavage sites (Figure 7A, gray boxes), as predicted by *in vitro* studies and comparison to known substrate cleavage sites (Figure 7A, black boxes). However, proteins found not to be processed by BACE1 also contain similar potential cleavage sites (Figure 7B). Therefore, BACE1 requires other permissive factors that are presently unclear to initiate substrate cleavage.

## Discussion

For its role in APP processing and Aβ generation, BACE1 is a central actor in the pathogenesis of Alzheimer's disease, and β-secretase inhibition has become a therapeutic goal. However, relatively little is known about the normal functions of BACE1 and how prolonged enzyme inhibition would affect the cellular processes it regulates. In this study, we have used unbiased and quantitative proteomic methods to identify proteins shed by BACE1 in two human epithelial cell lines. We have added over 60 putative β-secretase substrates to the previously small list of known BACE1 targets. The vast majority of the substrates have a type I topology, though some type II and GPI-linked proteins can also be cleaved.

To validate the proteomics findings, we either probed for endogenously expressed proteins or stably transfected a tagged cDNA encoding the putative substrate into a cell culture model. We examined putative substrates of type I, type II or GPI-linked topology identified by our screen, and in every case, the identified substrate was indeed confirmed to be processed by β-secretase. In addition to ectodomain shedding, the levels of the membrane-bound full-length protein were typically decreased with β-secretase activity, as expected. We validated both substrates of high confidence, in which over 100 peptides had been identified by mass spectrometry, and low confidence, in which as little as one peptide was identified. These results suggest that the remaining putative β-secretase substrates emerging from our screen are indeed subject to BACE1 cleavage, and future investigation will shed light on the biological significance of their processing.

Previously, we used similar proteomic methods to identify novel γ-secretase substrates [22]. In comparison to the former study, we have identified far more putative β-secretase substrates, and the likely reasons lie both in the less complex subcellular fractionation (secreted proteins versus membrane-bound proteins) used here and the larger sizes of the proteins subjected to analysis (ectodomains versus CTFs, thus offering more peptides for detection). The identification of APLP1, which is expressed at very low levels in the epithelial cells we tested, provides a clear example of the sensitivity of these methods. Considering that the large majority of putative β-secretase substrates are type I transmembrane proteins, it is highly likely that the residual membrane-anchored CTFs produced after ectodomain shedding are processed by γ-secretase to generate secreted Aβ-like peptides and a soluble intracellular domain. If true, our findings would substantially expand the list of known γ-secretase substrates.

In addition to the novel β-secretase substrates it identified, our unbiased proteomic method also revealed other proteins that are elevated by BACE1 overexpression but are unlikely to be directly processed by the protease. We found that the majority of these proteins are soluble lysosomal enzymes. The identification of IGF2R/M6PR ectodomain shedding by BACE1 provides a plausible mechanistic explanation for the presence of the extracellular lysosomal enzymes (see Table S2). This cellular phenotype, in which lysosomal proteins accumulate in the extracellular space, is mechanistically similar to the autosomal recessive human disorder, I-cell disease (mucolipidosis II), in which mutations in N-acetylglucosamine 1-phosphotransferase disrupt normal mannose-6-phosphate labeling of lysosomal proteins, preventing their receptor binding and leading to their secretion. In the analogous case of cellular BACE1 overexpression, elevated IGF2R shedding reduces the number of intact receptors able to mediate lysosomal transport and/or promotes the co-shedding of the IGF2R ectodomain with bound lysosomal proteins, thus increasing lysosomal protein secretion. Only four other non-membrane bound proteins were found to be elevated in conditioned medium due to BACE1 overexpression in our screen, and this may well have occurred through similar, but less clear, mechanisms of mistrafficking or co-shedding with an actual substrate.

The mechanism by which BACE1 selects its substrates has remained unclear despite over a decade of examination. BACE1 is related to other aspartyl proteases, including renin, cathepsin D and pepsin, and shows ∼50% sequence identity with the homologous BACE2 protein. There is a high degree of BACE1 sequence conservation among mammalian species [18]. However, except for BACE2, there is little similarity between BACE1 and other aspartyl proteases in terms of substrate profiles and inhibitor sensitivity. *In vitro* experiments with peptide libraries have shown BACE1 to prefer hydrophobic residues at P1 and P3, to accept polar residues at positions P2' and P1, and to display very low catalytic efficiency, even towards optimized peptide substrates [17]. Molecular modeling studies have identified a hydrophobic pocket that may bind to the P1 hydrophobic residue, and Arg296 also may form a salt bridge with a charged P1' residue, which may help explain BACE1's unique substrate preferences [18].

Another approach to determining substrate specificity has been to make modified APP constructs and monitor alterations in β-secretase processing [16]. These studies showed that, at least for APP, cleavage requires a membrane anchor and most mutations around the scissile bond decrease processing. Almost the entire APP ectodomain can be deleted without changing cleavage efficiency, and the cleavage site can tolerate shifts of at least five amino acids closer to the membrane. In other studies, introduction of the seven amino acids flanking the APP cleavage site into the juxtamembrane domain of a non-substrate permitted β-secretase cleavage of the hybrid protein [26].

These results all suggest that BACE1 has relatively loose sequence specificity and that regions outside the cleavage site may not play a critical role in substrate selection. However, in candidate-based approaches, the enzyme has been unable to process many type I transmembrane proteins known to be shed by the more promiscuous α-secretases (metalloproteases that include ADAMs and MMPs) [27], [28]. Analysis of the primary sequences of BACE1 substrates reveals potential cleavage sites, but similar sequences are also found in non-substrates (Figure 7), indicating that other factors are required for β-secretase processing. These factors may include accessibility of the cleavage site, sequestration of the substrate through incorporation into larger complexes, conformational change after ligand binding, and subcellular localization. Our identification of many new putative substrates should enable experiments to understand better the mechanisms underlying BACE1 substrate selection and what role β-secretase processing plays in modulating a particular substrate's function. Taken together, the findings presented here should enhance our understanding of the normal cellular functions to which BACE1 contributes and improve our search for potential adverse events when considering this protease as a therapeutic target.

## Materials and Methods

### Cell Culture and Treatments

The epithelial cell lines HeLa (ATCC) and human embryonic kidney (HEK) 293-FT (Invitrogen) were grown in Dulbecco's modified Eagle's medium containing 10% FBS, 2 mM L-glutamine, 100 µg/ml penicillin, and 100 µg/ml streptomycin. Transfections were performed with Fugene 6 (Roche Applied Sciences). Stable cell lines were generated by transduction with lentivirus containing the cDNAs of interest, as previously described [6]. Cells were treated with the β-secretase inhibitor C3 (3–6 µM, BACE inhibitor IV), the metalloprotease inhibitor GM6001 (10–20 µM) or the γ-secretase inhibitor DAPT (10 µM) for 18 to 20 hr in Opti-MEM I (Invitrogen). All drugs were purchased from Calbiochem.

### SILAC and LC-MS/MS

HEK and HeLa cell lines expressing either human BACE1 or an empty vector control were propagated for six doublings in DMEM lacking l-lysine and l-arginine (Invitrogen), and supplemented with 10% dialyzed fetal bovine serum (FBS) (Calbiochem), antibiotics, and either ^12^C^14^N arginine plus ^12^C^14^N lysine (“light”) or ^13^C^15^N arginine plus ^13^C^15^N lysine (“heavy”) (Cambridge Isotope Laboratories). Cells overexpressing BACE1 were grown under heavy labeling conditions, and control cells under light. Once labeled, the confluent cells were extensively washed and then treated with serum-free medium containing 20 µM GM6001 for 20 hr to inhibit α-secretases. Conditioned medium from this treatment was collected, floating cellular debris removed by centrifugation, and a small aliquot was analyzed by Western blotting.

Equal volumes of conditioned medium from BACE1 overexpressing and control cells were combined and concentrated approximately 200-fold using Centricon filter devices with a 3 kDa cutoff (Millipore). Approximately one hundred micrograms of protein from the concentrated media were run on an 4–12% Bis-Tris SDS-PAGE gel, stained with Coomassie Blue, divided into ten horizontal gel slices by molecular weight, and subject to in-gel digestion with trypsin (see Figure S1 for a schematic).

Liquid chromatography tandem mass spectrometry (LC-MS/MS) was performed using an LTQ Orbitrap hybrid mass spectrometer (Thermo Scientific). Resulting MS/MS spectra were matched to a composite target-decoy [55] human sequence database [56], using both SEQUEST and Mascot search engines. An in-house algorithm was used to select confident peptide identifications with an estimated false discovery rate less than 1%. Confident peptide identifications were then subjected to Vista, an automated software suite which measures the relative abundance of light and heavy isotopic peptide pairs [57], [58]. Proteins containing peptides with at least 65% of the total (light plus heavy) signal derived from the BACE1 (heavy) condition were considered as putative substrates. This threshold value is equivalent to a 1.857-fold (0.65/0.35) increase in peptide abundance.

### Cloning

Full-length cDNAs were obtained from the NIH Mammalian Gene Collection unless otherwise described. The LRIG2, LRIG3 and GOLIM4 cDNA were from mouse, and all other cDNAs were human in origin. Type I transmembrane and GPI-linked proteins were N-terminally tagged with the FLAG epitope (DYKDDDDK) by inserting the sequence encoding FLAG into primers and using an overlapping PCR method to generate the full-length tagged construct as previously described [22]. Epitope tags were inserted at least 5 amino acids downstream of the predicted signal peptide cleavage site. Type II transmembrane proteins were C-terminally tagged by inserting the FLAG sequence upstream of the stop codon, thus adding the epitope tag to the ectodomain. An extreme C-terminal HA tag (YPYDVPDYA) was also added to semaphorin 4C by identical methods. BACE1 was C-terminally myc-tagged. All expression constructs were verified by DNA sequencing.

### Immunoblotting

Cells were lysed in 50 mM Tris-HCl (pH 7.4), 1% NP-40, protease inhibitor cocktail (Roche Applied Sciences), 2 mM 1,10-phenanthroline and 5 mM EDTA. Lysates were centrifuged at 1,000 *g* for 10 min to remove nuclei. Protein concentrations were determined using a bicinchoninic acid-based assay (Pierce Biotechnology). Conditioned media were collected, cellular debris removed by centrifugation, and 500 µl of each medium was concentrated using a Microcon filter device (Millipore) with a membrane cutoff of 30 kDa. Samples were then subjected to SDS-PAGE and Western blotting. APP was detected using the polyclonal antibody C9 (1∶1,000) [53], CT20 (1∶1,000, Calbiochem), or the ectodomain directed antibody 22C11 (1∶1,000, Chemicon); nicastrin with anti-nicastrin (1∶1,000, BD Biosciences); IGF2R with HPA011332 (1∶1,000, Sigma); integrin beta-1 with 4706 (1∶1,000, Cell Signaling); APLP1 with 1NT (1∶1,000) [46]; BACE1 with Z-183 (1∶1,000, Santa Cruz Biotechnology, Inc.) or anti-myc 9E10 (1∶1,000; Santa Cruz Biotechnology, Inc.); ACE with H-170 (1∶1,000, Santa Cruz Biotechnology, Inc); HA tag with 3F10 (1∶1,000, Roche Applied Sciences); and FLAG tag with M2 (1∶1,000, Sigma). Western blots were probed with anti-mouse, anti-rabbit or anti-rat secondary antibodies (1∶10,000, Rockland Immunochemicals) and detected using the Odyssey infrared imaging system (LI-COR Biosciences). Immunoblots shown are representative of at least three experiments. Immunoreactive proteins were quantified using Odyssey Software v1.2, and the data were analyzed using a one-way analysis of variance with Tukey post-hoc comparison or a two-tailed Student *t*-test, where appropriate. Calculated comparisons of *p* < 0.05 were considered significant. All reported values represent the means ± standard error of the mean (SEM).

## Supporting Information

Table S1 Expanded information on putative β-secretase substrates.
(0.05 MB XLS)

Table S2 Putative non-substrate proteins elevated with BACE1 expression.
(0.03 MB XLS)

Figure S1 SILAC and LC-MS/MS approach to identifying proteins differentially shed between BACE1 and control cell lines. BACE1 and control cells were grown in the presence of heavy (BACE1) or light (control) lysine and arginine. Once labeled, the cells were incubated in serum-free medium, and the conditioned medium was collected. (A) APPs levels were evaluated in the resulting medium (the HEK line is shown), and equal volumes of the collected medium from BACE1 and control cells were combined. (B) The combined media were concentrated 200-fold, and approximately 100 µg of protein was separated on an SDS-PAGE gel. The gel was cut into 10 horizontal regions of approximately equal protein abundance. (C) Proteins were trypsinized and subject to LC-MS/MS analysis. Putative BACE1 substrates elevated in conditioned medium would be expected to show an increased relative abundance of peptides, which being labeled with heavy amino acids would have a predictably increased m/z ratio compared to the control condition. A graphical example of data from the proteomics screen is shown in D and E. (D) MS spectra of the APP peptide LEVPTDGNAGLLAEPQIAMFCGR, with the red vertical line indicating the beginning of the light spectra and the blue line indicating the heavy (BACE1) spectra. (E) Relative abundance of APP peptides in heavy (BACE1, blue) and light (control, red) conditions. The area underneath each of these curves was used to calculate the ratio of BACE1 peptides to total peptides.
(1.00 MB TIF)

Figure S2 Mapping of identified peptides to putative β-secretase substrates.
(0.29 MB PDF)
