# Supplementary figures and images for: Identification of β-Secretase (BACE1) Substrates Using Quantitative Proteomics
Source: PLoS One. 2009 Dec 29;4(12):e8477. doi: 10.1371/journal.pone.0008477 (PMC2793532; doi:10.1371/journal.pone.0008477)

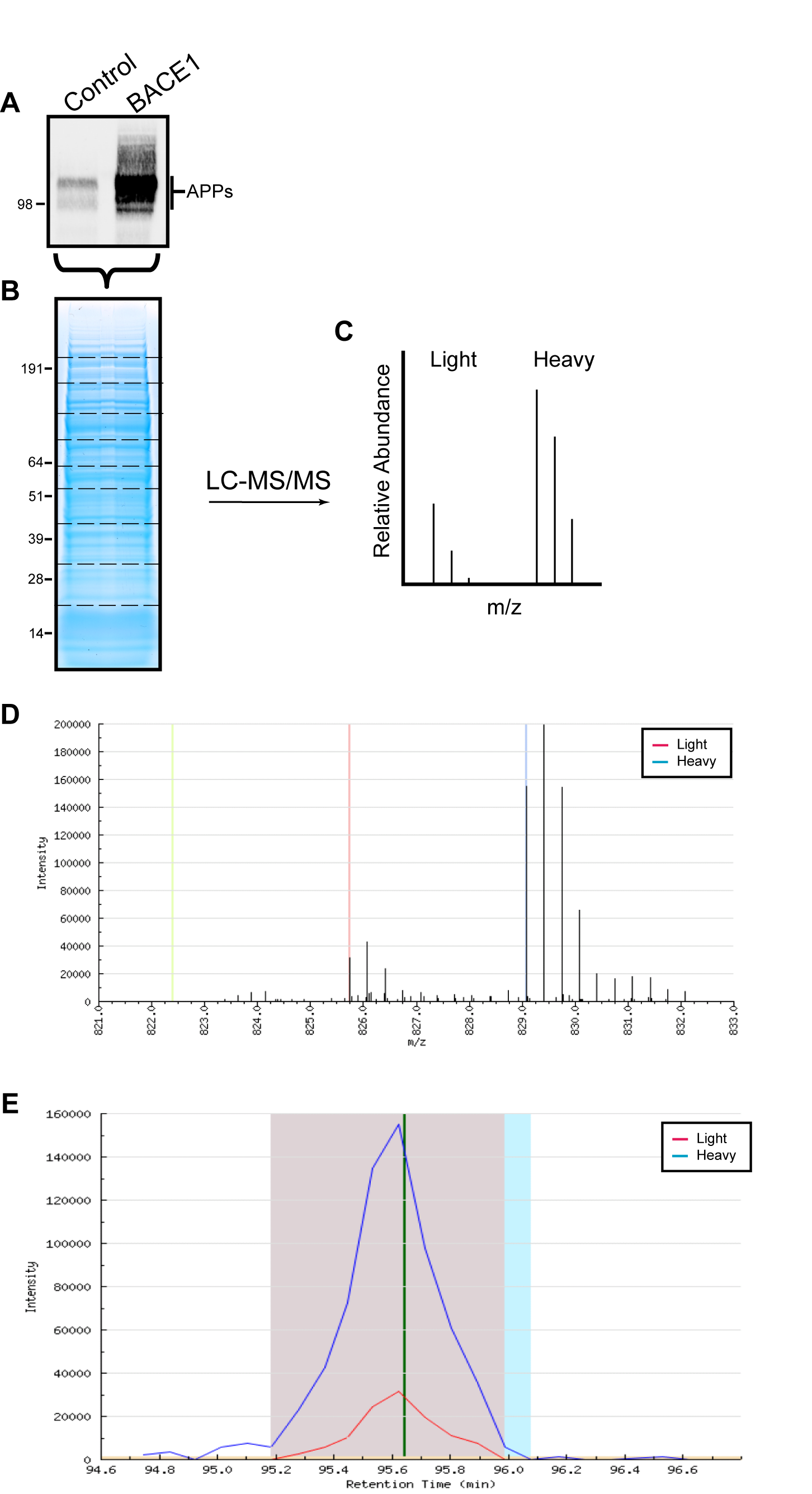

Supplement: Figure S1 — SILAC and LC-MS/MS approach to identifying proteins differentially shed between BACE1 and control cell lines. BACE1 and control cells were grown in the presence of heavy (BACE1) or light (control) lysine and arginine. Once labeled, the cells were incubated in serum-free medium, and the conditioned medium was collected. (A) APPs levels were evaluated in the resulting medium (the HEK line is shown), and equal volumes of the collected medium from BACE1 and control cells were combined. (B) The combined media were concentrated 200-fold, and approximately 100 µg of protein was separated on an SDS-PAGE gel. The gel was cut into 10 horizontal regions of approximately equal protein abundance. (C) Proteins were trypsinized and subject to LC-MS/MS analysis. Putative BACE1 substrates elevated in conditioned medium would be expected to show an increased relative abundance of peptides, which being labeled with heavy amino acids would have a predictably increased m/z ratio compared to the control condition. A graphical example of data from the proteomics screen is shown in D and E. (D) MS spectra of the APP peptide LEVPTDGNAGLLAEPQIAMFCGR, with the red vertical line indicating the beginning of the light spectra and the blue line indicating the heavy (BACE1) spectra. (E) Relative abundance of APP peptides in heavy (BACE1, blue) and light (control, red) conditions. The area underneath each of these curves was used to calculate the ratio of BACE1 peptides to total peptides. (1.00 MB TIF) [file pone.0008477.s003.tif]
