# Supplementary material for: Identification of β-Secretase (BACE1) Substrates Using Quantitative Proteomics
Source: PLoS One. 2009 Dec 29;4(12):e8477. doi: 10.1371/journal.pone.0008477 (PMC2793532; doi:10.1371/journal.pone.0008477)
Supplement: Figure S2 — Mapping of identified peptides to putative β-secretase substrates. (0.29 MB PDF) [file pone.0008477.s004.pdf]

Figure S2. Mapping of identified peptides to putative β-secretase substrates. For each protein, the table lists the identified peptides, the cell line where the peptide was identified, how many PSMs were found that correspond to the peptide, and the ratio indicating the accumulation of the peptide in the BACE1 condition. Transmembrane domains and GPI-liked residues are indicated in yellow. Identified peptides are colored in red.

Type I Transmembrane Proteins

Protein	Peptide	Cell Line	PSMs	Ratio (BACE/total)
APP	MDAEFRHDSGY	HEK	59	0.96
APP	AMISRWYFDVTEGK	HeLa	50	0.93
APP	LEVPTDGNAGLLAEPQIAMFCGR	HEK	30	0.89
APP	LEVPTDGNAGLLAEPQIAMFCGR	HeLa	18	0.82
APP	HVFNMLK	HEK	8	0.97
APP	LEVPTDGNAGLLAEPQIAMFCGR	HEK	2	0.66
APP	STNLHDYGMLLPCGIDKFR	HEK	1	0.96
APP	LALENYITALQAVPPRPR	HEK	1	0.94

>IPI:IPI00006608.1 Homo sapiens (Human) ISOFORM APP770 OF AMYLOID BETA A4 PROTEIN PRECURSOR (FRAGMENT). [MASS=86943]

MLPGLALLLL AAWTARALEV PTDGNAGLLA EPQIAMFCGR LNMHMNVQNG KWDSDPSGTK TCIDTKEGIL
QYCQEVYPEL QITNVVEANQ PVTIQNWCKR GRKQCKTHPH FVIPYRCLVG EFVSDALLVP DKCKFLHQER
MDVCETHLHW HTVAKETCSE KSTNLHDYGM LLPCGIDKFR GVEFVCCPLA EESDNVDSAD AEEDDSDVWW
GGADTDYADG SEDKVVEVAE EEEVAEVEEE EADDDEDDED GDEVEEEAEE PYEEATERTT SIATTTTTTT
ESVEEVVREV CSEQAETGPC RAMISRWYFD VTEGKCAPFF YGGCGGNRNN FDTEEYCMAV CGSAMSQSLL
KTTQEPLARD PVKLPTTAAS TPDAVDKYLE TPGDENEHAH FQKAKERLEA KHRERMSQVM REWEEAERQA
KNLPKADKKA VIQHFQEKVE SLEQEAANER QQLVETHMAR VEAMLNDRRR LALENYITAL QAVPPRPRHV
FNMLKKYVRA EQKDRQHTLK HFEHVRMVDP KKAAQIRSQV MTHLRVIYER MNQSLSLLYN VPAVAEEIQD
EVDELLQKEQ NYSDDVLANM ISEPRISYGN DALMPSLTET KTTVELLPVN GEFSLDDLQP WHSFGADSVP
ANTENEVEPV DARPAADRGL TTRPGSGLTN IKTEEISEVK MDAEFRHDSG YEVHHQKLVF FAEDVGSNKG
AIIGLMVGGV VIATVIVITL VMLKKKQYTS IHHGVVEVDA AVTPEERHLS KMQQNGYENP TYKFFEQMQN

Protein	Peptide	Cell Line	PSMs	Ratio (BACE/total)
APLP2	RMALENYLAALQSD	HEK	57	0.98
APLP2	HYQHVLAVD	HEK	45	0.98
APLP2	VDENMVIDETL	HEK	35	0.96
APLP2	LNMHVNIQTGKWEPDPTGTK	HEK	12	0.99
APLP2	AERQTLIQHFQAMVK	HeLa	7	0.96
APLP2	GSGVGEQDGGLIGAEEK	HEK	5	0.82
APLP2	KGSGVGEQDGGLIGAEEK	HeLa	2	0.98
APLP2	LNMHVNIQTGKWEPDPTGTK	HeLa	1	1.00
APLP2	VGGLEEERESVGPLR	HEK	1	0.95

>IPI:IPI00031030.1 Homo sapiens (Human) ISOFORM 1 OF AMYLOID-LIKE PROTEIN 2 PRECURSOR. [MASS=86956]

MAATGTAAAA ATGRLLLLLL VGLTAPALAL AGYIEALAAN AGTGFAVAEP QIAMFCGKLN MHVNIQTGKW
EPDPTGTKSC FETKEEVLQY CQEMYPELQI TNVMEANQRV SIDNWCRRDK KQCKSRFVTP FKCLVGEFVS
DVLLVPEKCQ FFHKERMEVC ENHQHWHTVV KEACLTQGMT LYSYGMLLPC GVDQFHGTEY VCCPQTKIIG
SVSKEEEEED EEEEEEEDEE EDYDVYKSEF PTEADLEDFT EAAVDEDDED EEEGEEVVED RDYYYDTFKG
DDYNEENPTE PGSDGTMSDK EITHDVKAVC SQEAMTGPCR AVMPRWYFDL SKGKCVRFIY GGCGGNRNNF

ESEDYCMAVC KAMIPPTPLP TNDVDVYFET SADDNEHARF QKAKEQLEIR HRNRMDRVKK EWEEAELQAK
NLPKAERQTL IQHFQAMVKA LEKEAASEKQ QLVETHLARV EAMLNDRRRM ALENYLAALQ SDPPRPHRIL
QALRRYVRAE NKDRLHTIRH YQHVLAVDPE KAAQMKSQVM THLHVIEERR NQSLSLLYKV PYVAQEIQEE
IDELLQEQRA DMDQFTASIS ETPVDVRVSS EESEEIPPFH PFHPFPALPE NEDTQPELYH PMKKGSGVGE
QDGGLIGAEE KVINSKNKVD ENMVIDETLD VKEMIFNAER VGGLEEERES VGPLREDFSL SSSALIGLLV
IAVAIATVIV ISLVMLRKRQ YGTISHGIVE VDPMLTPEER HLNKMQNHGY ENPTYKYLEQ MQI

Protein	Peptide	Cell Line	PSMs	Ratio (BACE/total)
APLP1	GFPFHSSEIQ	HEK	6	0.89
APLP1	VLLALRR	HEK	2	0.84
APLP1	VLEYCR	HeLa	1	0.90
APLP1	GFPFHSSEIQR	HeLa	1	0.95

>IPI:IPI00020012.2 Homo sapiens (Human) AMYLOID-LIKE PROTEIN 1 PRECURSOR. [MASS=72176]
MGPASPAARG LSRRPGQPPL PLLLPLLLLL LRAQPAIGSL AGGSPGAAEA PGSAQVAGLC GRLTLHRDLR
TGRWEPDPQR SRRCLRDPQR VLEYCRQMYP ELQIARVEQA TQAIPMERWC GGSRSGSCAH PHHQVVPFRC
LPGEFVSEAL LVPEGCRFLH QERMDQCESS TRRHQEAQEA CSSQGLILHG SGMLLPCGSD RFRGVEYVCC
PPPGTPDPSG TAVGDPSTRS WPPGSRVEGA EDEEEEESFP QPVDDYFVEP PQAEEEEETV PPPSSHTLAV
VGKVTPTPRP TDGVDIYFGM PGEISEHEGF LRAKMDLEER RMRQINEVMR EWAMADNQSK NLPKADRQAL
NEHFQSILQT LEEQVSGERQ RLVETHATRV IALINDQRRA ALEGFLAALQ ADPPQAERVL LALRRYLRAE
QKEQRHTLRH YQHVAAVDPE KAQQMRFQVH THLQVIEERV NQSLGLLDQN PHLAQELRPQ IQELLHSEHL
GPSELEAPAP GGSSEDKGGL QPPDSKDDTP MTLPKGSTEQ DAASPEKEKM NPLEQYERKV NASVPRGFPF
HSSEIQRDEL APAGTGVSRE AVSGLLIMGA GGGSLIVLSM LLLRRKKPYG AISHGVVEVD PMLTLEEQQL
RELQRHGYEN PTYRFLEERP

Protein	Peptide	Cell Line	PSMs	Ratio(BACE/total)
IGF2R	VVSSCQEKREPQGFHK	HEK	104	0.90
IGF2R	LTYENGLLK	HeLa	26	0.88
IGF2R	SYEECIIESR	HEK	3	0.91
IGF2R	TSGEGGCFYEFEWHTAAACVLSK	HEK	2	0.82

>IPI:IPI00289819.4 Homo sapiens (Human) CATION-INDEPENDENT MANNOSE-6-PHOSPHATE RECEPTOR PRECURSOR. [MASS=274276] Insulin-like growth factor 2 receptor
MGAAAGRSPH LGPAPARRPQ RSLLLLQLLL LVAAPGSTQA QAAPFPELCS YTWEAVDTKN NVLYKINICG
SVDIVQCGPS SAVCMHDLKT RTYHSVGDSV LRSATRSLLE FNTTVSCDQQ GTNHRVQSSI AFLCGKTLGT
PEFVTATECV HYFEWRTTAA CKKDIFKANK EVPCYVFDEE LRKHDLNPLI KLSGAYLVDD SDPDTSLFIN
VCRDIDTLRD PGSQLRACPP GTAACLVRGH QAFDVGQPRD GLKLVRKDRL VLSYVREEAG KLDFCDGHSP
AVTITFVCPS ERREGTIPKL TAKSNCRYEI EWITEYACHR DYLESKTCSL SGEQQDVSID LTPLAQSGGS
SYISDGKEYL FYLNVCGETE IQFCNKKQAA VCQVKKSDTS QVKAAGRYHN QTLRYSDGDL TLIYFGGDEC
SSGFQRMSVI NFECNKTAGN DGKGTPVFTG EVDCTYFFTW DTEYACVKEK EDLLCGATDG KKRYDLSALV
RHAEPEQNWE AVDGSQTETE KKHFFINICH RVLQEGKARG CPEDAAVCAV DKNGSKNLGK FISSPMKEKG
NIQLSYSDGD DCGHGKKIKT NITLVCKPGD LESAPVLRTS GEGGCFYEFE WHTAAACVLS KTEGENCTVF
DSQAGFSFDL SPLTKKNGAY KVETKKYDFY INVCGPVSVS PCQPDSGACQ VAKSDEKTWN LGLSNAKLSY
YDGMIQLNYR GGTPYNNERH TPRATLITFL CDRDAGVGFP EYQEEDNSTY NFRWYTSYAC PEEPLECVVT
DPSTLEQYDL SSLAKSEGGL GGNWYAMDNS GEHVTWRKYY INVCRPLNPV PGCNRYASAC QMKYEKDQGS
FTEVVSISNL GMAKTGPVVE DSGSLLLEYV NGSACTTSDG RQTTYTTRIH LVCSRGRLNS HPIFSLNWEC
VVSFLWNTEA ACPIQTTTDT DQACSIRDPN SGFVFNLNPL NSSQGYNVSG IGKIFMFNVC GTMPVCGTIL
GKPASGCEAE TQTEELKNWK PARPVGIEKS LQLSTEGFIT LTYKGPLSAK GTADAFIVRF VCNDDVYSGP
LKFLHQDIDS GQGIRNTYFE FETALACVPS PVDCQVTDLA GNEYDLTGLS TVRKPWTAVD TSVDGRKRTF
YLSVCNPLPY IPGCQGSAVG SCLVSEGNSW NLGVVQMSPQ AAANGSLSIM YVNGDKCGNQ RFSTRITFEC
AQISGSPAFQ LQDGCEYVFI WRTVEACPVV RVEGDNCEVK DPRHGNLYDL KPLGLNDTIV SAGEYTYYFR
VCGKLSSDVC PTSDKSKVVS SCQEKREPQG FHKVAGLLTQ KLTYENGLLK MNFTGGDTCH KVYQRSTAIF

FYCDRGTQRP VFLKETSDCS YLFEWRTQYA CPPFDLTECS FKDGAGNSFD LSSLSRYSDN WEAITGTGDP
EHYLINVCKS LAPQAGTEPC PPEAAACLLG GSKPVNLGRV RDGPQWRDGI IVLKYVDGDL CPDGIRKKST
TIRFTCSESQ VNSRPMFISA VEDCEYTFAW PTATACPMKS NEHDDCQVTN PSTGHLFDLS SLSGRAGFTA
AYSEKGLVYM SICGENENCP PGVGACFGQT RISVGKANKR LRYVDQVLQL VYKDGSPCPS KSGLSYKSVI
SFVCRPEAGP TNRPMLISLD KQTCTLFFSW HTPLACEQAT ECSVRNGSSI VDLSPLIHRT GGYEAYDESE
DDASDTNPDF YINICQPLNP MHGVPCPAGA AVCKVPIDGP PIDIGRVAGP PILNPIANEI YLNFESSTPC
LADKHFNYTS LIAFHCKRGV SMGTPKLLRT SECDFVFEWE TPVVCPDEVR MDGCTLTDEQ LLYSFNLSSL
STSTFKVTRD SRTYSVGVCT FAVGPEQGGC KDGGVCLLSG TKGASFGRLQ SMKLDYRHQD EAVVLSYVNG
DRCPPETDDG VPCVFPFIFN GKSYEECIIE SRAKLWCSTT ADYDRDHEWG FCRHSNSYRT SSIIFKCDED
EDIGRPQVFS EVRGCDVTFE WKTKVVCPPK KLECKFVQKH KTYDLRLLSS LTGSWSLVHN GVSYYINLCQ
KIYKGPLGCS ERASICRRTT TGDVQVLGLV HTQKLGVIGD KVVVTYSKGY PCGGNKTASS VIELTCTKTV
GRPAFKRFDI DSCTYYFSWD SRAACAVKPQ EVQMVNGTIT NPINGKSFSL GDIYFKLFRA SGDMRTNGDN
YLYEIQLSSI TSSRNPACSG ANICQVKPND QHFSRKVGTS DKTKYYLQDG DLDVVFASSS KCGKDKTKSV
SSTIFFHCDP LVEDGIPEFS HETADCQYLF SWYTSAVCPL GVGFDSENPG DDGQMHKGLS ERSQAVGAVL
SLLLVALTCC LLALLLYKKE RRETVISKLT TCCRRSSNVS YKYSKVNKEE ETDENETEWL MEEIQLPPPR
QGKEGQENGH ITTKSVKALS SLHGDDQDSE DEVLTIPEVK VHSGRGAGAE SSHPVRNAQS NALQEREDDR
VGLVRGEKAR KGKSSSAQQK TVSSTKLVSF HDDSDEDLLH I

Protein	Peptide	Cell Line	PSMs	Ratio(BACE/total)
CBPD	IEWIRR	HEK	67	0.95
CBPD	NKFVLSGNLHGGSVVASYPFDDSPEHKATGIYSK	HEK	23	0.82
CBPD	FVLSGNLHGGSVVASYPFDDSPEHK	HEK	1	0.84

>IPI:IPI00027078.2 Homo sapiens (Human) CARBOXYPEPTIDASE D PRECURSOR. [MASS=152915]

MASGRDERPP WRLGRLLLLM CLLLLGSSAR AAHIKKAEAT TTTTSAGAEA AEGQFDRYYH EEELESALRE
AAAAGLPGLA RLFSIGRSVE GRPLWVLRLT AGLGSLIPEG DAGPDAAGPD AAGPLLPGRP QVKLVGNMHG
DETVSRQVLI YLARELAAGY RRGDPRLVRL LNTTDVYLLP SLNPDGFERA REGDCGFGDG GPSGASGRDN
SRGRDLNRSF PDQFSTGEPP ALDEVPEVRA LIEWIRRNKF VLSGNLHGGS VVASYPFDDS PEHKATGIYS
KTSDDEVFKY LAKAYASNHP IMKTGEPHCP GDEDETFKDG ITNGAHWYDV EGGMQDYNYV WANCFEITLE
LSCCKYPPAS QLRQEWENNR ESLITLIEKV HIGVKGFVKD SITGSGLENA TISVAGINHN ITTGRFGDFY
RLLVPGTYNL TVVLTGYMPL TVTNVVVKEG PATEVDFSLR PTVTSVIPDT TEAVSTASTV AIPNILSGTS
SSYQPIQPKD FHHHHFPDME IFLRRFANEY PNITRLYSLG KSVESRELYV MEISDNPGVH EPGEPEFKYI
GNMHGNEVVG RELLLNLIEY LCKNFGTDPE VTDLVHNTRI HLMPSMNPDG YEKSQEGDSI SVIGRNNSNN
FDLNRNFPDQ FVQITDPTQP ETIAVMSWMK SYPFVLSANL HGGSLVVNYP FDDDEQGLAT YSKSPDDAVF
QQIALSYSKE NSQMFQGRPC KNMYPNEYFP HGITNGASWY NVPGGMQDWN YLQTNCFEVT IELGCVKYPL
EKELPNFWEQ NRRSLIQFMK QVHQGVRGFV LDATDGRGIL NATISVAEIN HPVTTYKTGD YWRLLVPGTY
KITASARGYN PVTKNVTVKS EGAIQVNFTL VRSSTDSNNE SKKGKGASSS TNDASVPTTK EFETLIKDLS
AENGLESLML RSSSNLALAL YRYHSYKDLS EFLRGLVMNY PHITNLTNLG QSTEYRHIWS LEISNKPNVS
EPEEPKIRFV AGIHGNAPVG TELLLALAEF LCLNYKKNPA VTQLVDRTRI VIVPSLNPDG RERAQEKDCT
SKIGQTNARG KDLDTDFTNN ASQPETKAII ENLIQKQDFS LSVALDGGSM LVTYPYDKPV QTVENKETLK
HLASLYANNH PSMHMGQPSC PNKSDENIPG GVMRGAEWHS HLGSMKDYSV TYGHCPEITV YTSCCYFPSA
ARLPSLWADN KRSLLSMLVE VHKGVHGFVK DKTGKPISKA VIVLNEGIKV QTKEGGYFHV LLAPGVHNII
AIADGYQQQH SQVFVHHDAA SSVVIVFDTD NRIFGLPREL VVTVSGATMS ALILTACIIW CICSIKSNRH
KDGFHRLRQH HDEYEDEIRM MSTGSKKSLL SHEFQDETDT EEETLYSSKH

Protein	Peptide	Cell Line	PSMs	Ratio(BACE/total)
HGFR (c-MET)	SAMCAFPIK	HEK	5	0.88
HGFR (c-MET)	HFQSCSQCLSAPPFVQCGWCHDK	HeLa	5	0.68
HGFR (c-MET)	ETSIFSYREDPIVYEIHPTK	HeLa	3	0.66

>IPI:IPI00029273.1 Homo sapiens (Human) ISOFORM 1 OF HEPATOCYTE GROWTH FACTOR RECEPTOR PRECURSOR. [MASS=155527]

**MKAPAVLAPG ILVLLFTLVQ RSNGECKEAL AKSEMNVNMK YQLPNFTAET PIQNVILHEH HIFLGATNYI
YVLNEEDLQK VAEYKTGPVL EHPDCFPCQD CSSKANLSGG VWKDNINMAL VVDTYYDDQL ISCGSVNRGT
CQRHVFPHNH TADIQSEVHC IFSPQIEEPS QCPDCVVSAL GAKVLSSVKD RFINFFVGNT INSSYFPDHP
LHSISVRRLK ETKDGFMFLT DQSYIDVLPE FRDSYPIKYV HAFESNNFIY FLTVQRETLD AQTFHTRIIR
FCSINSGLHS YMEMPLECIL TEKRKKRSTK KEVFNILQAA YVSKPGAQLA RQIGASLNDD ILFGVFAQSK
PDSAEPMDRS AMCAFPIKYV NDFFNKIVNK NNVRCLQHFY GPNHEHCFNR TLLRNSSGCE ARRDEYRTEF
TTALQRVDLF MGQFSEVLLT SISTFIKGDL TIANLGTSEG RFMQVVVSRS GPSTPHVNFL LDSHPVSPEV
IVEHTLNQNG YTLVITGKKI TKIPLNGLGC RHFQSCSQCL SAPPFVQCGW CHDKCVRSEE CLSGTWTQQI
CLPAIYKVFP NSAPLEGGTR LTICGWDFGF RRNNKFDLKK TRVLLGNESC TLTLSESTMN TLKCTVGPAM
NKHFNMSIII SNGHGTTQYS TFSYVDPVIT SISPKYGPMA GGTLLTLTGN YLNSGNSRHI SIGGKTCTLK
SVSNSILECY TPAQTISTEF AVKLKIDLAN RETSIFSYRE DPIVYEIHPT KSFISGGSTI TGVGKNLNSV
SVPRMVINVH EAGRNFTVAC QHRSNSEIIC CTTPSLQQLN LQLPLKTKAF FMLDGILSKY FDLIYVHNPV
FKPFEKPVMI SMGNENVLEI KGNDIDPEAV KGEVLKVGNK SCENIHLHSE AVLCTVPNDL LKLNSELNIE
WKQAISSTVL GKVIVQPDQN FTGLIAGVVS ISTALLLLLG FFLWLKKRKQ IKDLGSELVR YDARVHTPHL
DRLVSARSVS PTTEMVSNES VDYRATFPED QFPNSSQNGS CRQVQYPLTD MSPILTSGDS DISSPLLQNT
VHIDLSALNP ELVQAVQHVV IGPSSLIVHF NEVIGRGHFG CVYHGTLLDN DGKKIHCAVK SLNRITDIGE
VSQFLTEGII MKDFSHPNVL SLLGICLRSE GSPLVVLPYM KHGDLRNFIR NETHNPTVKD LIGFGLQVAK
GMKYLASKKF VHRDLAARNC MLDEKFTVKV ADFGLARDMY DKEYYSVHNK TGAKLPVKWM ALESLQTQKF
TTKSDVWSFG VVLWELMTRG APPYPDVNTF DITVYLLQGR RLLQPEYCPD PLYEVMLKCW HPKAEMRPSF
SELVSRISAI FSTFIGEHYV HVNATYVNVK CVAPYPSLLS SEDNADDEVD TRPASFWETS**

Protein	Peptide	Cell Line	PSMs	Ratio(BACE/total)
IL6R beta	EDGKGYWSDWSEEASGITYEDRPSK	HEK	6	0.97
IL6R beta	TVQLVWK	HEK	1	1.00
IL6R beta	APCITDWQQEDGTVHR	HeLa	1	0.97

>IPI:IPI00297124.1 Homo sapiens (Human) ISOFORM 1 OF INTERLEUKIN-6 RECEPTOR BETA CHAIN PRECURSOR. [MASS=103523]

**MLTLQTWVVQ ALFIFLTTES TGELLDPCGY ISPESPVVQL HSNFTAVCVL KEKCMDYFHV NANYIVWKTN
HFTIPKEQYT IINRTASSVT FTDIASLNIQ LTCNILTFGQ LEQNVYGITI ISGLPPEKPK NLSCIVNEGK
KMRCEWDGGR ETHLETNFTL KSEWATHKFA DCKAKRDTPT SCTVDYSTVY FVNIEVWVEA ENALGKVTSD
HINFDPVYKV KPNPPHNLSV INSEELSSIL KLTWTNPSIK SVIILKYNIQ YRTKDASTWS QIPPEDTAST
RSSFTVQDLK PFTEYVFRIR CMKEDGKGYW SDWSEEASGI TYEDRPSKAP SFWYKIDPSH TQGYRTVQLV
WKTLPPFEAN GKILDYEVTL TRWKSHLQNY TVNATKLTVN LTNDRYLATL TVRNLVGKSD AAVLTIPACD
FQATHPVMDL KAFPKDNMLW VEWTTPRESV KKYILEWCVL SDKAPCITDW QQEDGTVHRT YLRGNLAESK
CYLITVTPVY ADGPGSPESI KAYLKQAPPS KGPTVRTKKV GKNEAVLEWD QLPVDVQNGF IRNYTIFYRT
IIGNETAVNV DSSHTEYTLS SLTSDTLYMV RMAAYTDEGG KDGPEFTFTT PKFAQGEIEA IVVPVCLAFL
LTTLLGVLFC FNKRDLIKKH IWPNVPDPSK SHIAQWSPHT PPRHNFNSKD QMYSDGNFTD VSVVEIEAND
KKPFPEDLKS LDLFKKEKIN TEGHSSGIGG SSCMSSSRPS ISSSDENESS QNTSSTVQYS TVVHSGYRHQ
VPSVQVFSRS ESTQPLLDSE ERPEDLQLVD HVDGGDGILP RQQYFKQNCS QHESSPDISH FERSKQVSSV
NEEDFVRLKQ QISDHISQSC GSGQMKMFQE VSAADAFGPG TEGQVERFET VGMEAATDEG MPKSYLPQTV
RQGGYMPQ**

Protein	Peptide	Cell Line	PSMs	Ratio(BACE/total)
ROBO1	DGSPLDDKDER	HEK	25	0.95
ROBO1	AYLEVTDVIADRPPPVIR	HEK	1	0.81
ROBO1	TVTFQCEATGNPQPAIFWRR	HeLa	1	0.67
ROBO2A	NYDLSDLPGPPSKPQVTDVTK	HEK	1	1.00

>IPI:IPI00219798.1 Homo sapiens (Human) ISOFORM 1 OF ROUNDABOUT HOMOLOG 1 PRECURSOR. [MASS=180930]

MKWKHVPFLV MISLLSLSPN HLFLAQLIPD PEDVERGNDH GTPIPTSDND DNSLGYTGSR LRQEDFPPRI
VEHPSDLIVS KGEPATLNCK AEGRPTPTIE WYKGGERVET DKDDPRSHRM LLPSGSLFFL RIVHGRKSRP
DEGVYVCVAR NYLGEAVSHN ASLEVAILRD DFRQNPSDVM VAVGEPAVME CQPPRGHPEP TISWKKDGSP
LDDKDERITI RGGKLMITYT RKSDAGKYVC VGTNMVGERE SEVAELTVLE RPSFVKRPSN LAVTVDDSAE
FKCEARGDPV PTVRWRKDDG ELPKSRYEIR DDHTLKIRKV TAGDMGSYTC VAENMVGKAE ASATLTVQEP
PHFVVKPRDQ VVALGRTVTF QCEATGNPQP AIFWRREGSQ NLLFSYQPPQ SSSRFSVSQT GDLTITNVQR
SDVGYYICQT LNVAGSIITK AYLEVTDVIA DRPPPVIRQG PVNQTVAVDG TFVLSCVATG SPVPTILWRK
DGVLVSTQDS RIKQLENGVL QIRYAKLGDT GRYTCIASTP SGEATWSAYI EVQEFGVPVQ PPRPTDPNLI
PSAPSKPEVT DVSRNTVTLS WQPNLNSGAT PTSYIIEAFS HASGSSWQTV AENVKTETSA IKGLKPNAIY
LFLVRAANAY GISDPSQISD PVKTQDVLPT SQGVDHKQVQ RELGNAVLHL HNPTVLSSSS IEVHWTVDQQ
SQYIQGYKIL YRPSGANHGE SDWLVFEVRT PAKNSVVIPD LRKGVNYEIK ARPFFNEFQG ADSEIKFAKT
LEEAPSAPPQ GVTVSKNDGN GTAILVSWQP PPEDTQNGMV QEYKVWCLGN ETRYHINKTV DGSTFSVVIP
FLVPGIRYSV EVAASTGAGS GVKSEPQFIQ LDAHGNPVSP EDQVSLAQQI SDVVKQPAFI AGIGAACWII
LMVFSIWLYR HRKKRNGLTS TYAGIRKVPS FTFTPTVTYQ RGGEAVSSGG RPGLLNISEP AAQPWLADTW
PNTGNNHNDC SISCCTAGNG NSDSNLTTYS RPADCIANYN NQLDNKQTNL MLPESTVYGD VDLSNKINEM
KTFNSPNLKD GRFVNPSGQP TPYATTQLIQ SNLSNNMNNG SGDSGEKHWK PLGQQKQEVA PVQYNIVEQN
KLNKDYRAND TVPPTIPYNQ SYDQNTGGSY NSSDRGSSTS GSQGHKKGAR TPKVPKQGGM NWADLLPPPP
AHPPPHSNSE EYNISVDESY DQEMPCPVPP ARMYLQQDEL EEEEDERGPT PPVRGAASSP AAVSYSHQST
ATLTPSPQEE LQPMLQDCPE ETGHMQHQPD RRRQPVSPPP PPRPISPPHT YGYISGPLVS DMDTDAPEEE
EDEADMEVAK MQTRRLLLRG LEQTPASSVG DLESSVTGSM INGWGSASEE DNISSGRSSV SSSDGSFFTD
ADFAQAVAAA AEYAGLKVAR RQMQDAAGRR HFHASQCPRP TSPVSTDSNM SAAVMQKTRP AKKLKHQPGH
LRRETYTDDL PPPPVPPPAI KSPTAQSKTQ LEVRPVVVPK LPSMDARTDR SSDRKGSSYK GREVLDGRQV
VDMRTNPGDP REAQEQQNDG KGRGNKAAKR DLPPAKTHLI QEDILPYCRP TFPTSNNPRD PSSSSSMSSR
GSGSRQREQA NVGRRNIAEM QVLGGYERGE DNNEELEETE S

>IPI:IPI00385980.8 Homo sapiens (Human) ROBO2 ISOFORM A. [MASS=153120]

MARRHERVTR RMWTWAPGLL MMTVVFWGHQ GNGQGQGSRL RQEDFPPRIV EHPSDVIVSK GEPTTLNCKA
EGRPTPTIEW YKDGERVETD KDDPRSHRML LPSGSLFFLR IVHGRRSKPD EGSYVCVARN YLGEAVSRNA
SLEVALLRDD FRQNPTDVVV AAGEPAILEC QPPRGHPEPT IYWKKDKVRI DDKEERISIR GGKLMISNTR
KSDAGMYTCV GTNMVGERDS DPAELTVFER PTFLRRPINQ VVLEEEAVEF RCQVQGDPQP TVRWKKDDAD
LPRGRYDIKD DYTLRIKKTM STDEGTYMCI AENRVGKMEA SATLTVRAPP QFVVRPRDQI VAQGRTVTFP
CETKGNPQPA VFWQKEGSQN LLFPNQPQQP NSRCSVSPTG DLTITNIQRS DAGYYICQAL TVAGSILAKA
QLEVTDVLTD RPPPIILQGP ANQTLAVDGT ALLKCKATGD PLPVISWLKE GFTFPGRDPR ATIQEQGTLQ
IKNLRISDTG TYTCVATSSS GETSWSAVLD VTESGATISK NYDLSDLPGP PSKPQVTDVT KNSVTLSWQP
GTPGTLPASA YIIEAFSQSV SNSWQTVANH VKTTLYTVRG LRPNTIYLFM VRAINPQGLS DPSPMSDPVR
TQDISPPAQG VDHRQVQKEL GDVLVRLHNP VVLTPTTVQV TWTVDRQPQF IQGYRVMYRQ TSGLQATSSW
QNLDAKVPTE RSAVLVNLKK GVTYEIKVRP YFNEFQGMDS ESKTVRTTEE APSAPPQSVT VLTVGSYNST
SISVSWDPPP PDHQNGIIQE YKIWCLGNET RFHINKTVDA AIRSVIIGGL FPGIQYRVEV AASTSAGVGV
KSEPQPIIIG RRNEVVITEN NNSITEQITD VVKQPAFIAG IGGACWVILM GFSIWLYWRR KKRKGLSNYA
VTFQRGDGGL MSNGSRPGLL NAGDPSYPWL ADSWPATSLP VNNSNSGPNE IGNFGRGDVL PPVPGQGDKT
ATMLSDGAIY SSIDFTTKTS YNSSSQITQA TPYATTQILH SNSIHELAVD LPDPQWKSSI QQKTDLMGFG
YSLPDQNKGN NGGKGGKKKK NKNSSKPQKN NGSTWANVPL PPPPVQPLPG TELEHYAVEQ QENGYDSDSW
CPPLPVQTYL HQGLEDELEE DDDRVPTPPV RGVASSPAIS FGQQSTATLT PSPREEMQPM LQAHLDELTR
AYQFDIAKQT WHIQSNNQPP QPPVPPLGYV SGALISDLET DVADDDADDE EEALEIPRPL RALDQTPGSS
MDNLDSSVTG KAFTSSQRPR PTSPFSTDSN TSAALSQSQR PRPTKKHKGG RMDQQPALPH RREGMTDEEA
LVPYSKPSFP SPGGHSSSGT ASSKGSTGPR KTEVLRAGHQ RNASDLLDIG YMGSNSQGQF TGEL

Protein	Peptide	Cell Line	PSMs	Ratio(BACE/total)
SDK1	YFTMQVR	HEK	14	0.88
SDK2	LVVIALVGLIFILLLVFVLIIR	HEK	7	0.83
SDK2	LVVIALVGLIFILLLVFVLIIR	HeLa	1	1.00

>IPI:IPI00784490.1 Homo sapiens (Human) ISOFORM 1 OF PROTEIN SIDEKICK-1 PRECURSOR. [MASS=242076]

MARGARPSAA GGGGGGAEPP ERAGPGRPRG SPPGRARPSL APRPGPEPSR PRAAPETPGG DTAGAGRCGG
RRAAKLGPGR RGWWALLALQ LHLLRALAQD DVAPYFKTEP GLPQIHLEGN RLVLTCLAEG SWPLEFKWMR
DDSELTTYSS EYKYIIPSLQ KLDAGFYRCV VRNRMGALLQ RKSEVQVAYM GSFMDTDQRK TVSQGRAAIL
NLLPITSYPR PQVTWFREGH KIIPSNRIAI TLENQLVILA TTTSDAGAYY VQAVNEKNGE NKTSPFIHLS
IARDVGTPET MAPTIVVPPG NRSVVAGSSE TTLECIASAR PVEDLSVTWK RNGVRITSGL HSFGRHLTIS
NPTSADTGPY VCEAALPGSA FEPARATAFL FIIEPPYFTA EPESRISAEV EETVDIGCQA MGVPLPTLQW
YKDAISISRL QNPRYKVLAS GGLRIQKLRP EDSGIFQCFA SNEGGEIQTH TYLDVTNIAP VFTQRPVDTT
VTDGMTAILR CEVSGAPKPA ITWKRENHIL ASGSVRIPRF MLLESGGLQI APVFIQDAGN YTCYAANTEG
SLNASATLTV WNRTSIVHPP EDHVVIKGTT ATLHCGATHD PRVSLRYVWK KDNVALTPSS TSRIVVEKDG
SLLISQTWSG DIGDYSCEIV SEGGNDSRMA RLEVIELPHS PQNLLVSPNS SHSHAVVLSW VRPFDGNSPI
LYYIVELSEN SSPWKVHLSN VGPEMTGVTV SGLTPARTYQ FRVCAVNEVG RGQYSAETSR LMLPEEPPSA
PPKNIVASGR TNQSIMVQWQ PPPETEHNGV LRGYILRYRL AGLPGEYQQR NITSPEVNYC LVTDLIIWTQ
YEIQVAAYNG AGLGVFSRAV TEYTLQGVPT APPQNVQTEA VNSTTIQFLW NPPPQQFING INQGYKLLAW
PADAPEAVTV VTIAPDFHGV HHGHITNLKK FTAYFTSVLC FTTPGDGPPS TPQLVWTQED KPGAVGHLSF
TEILDTSLKV SWQEPLEKNG IITGYQISWE VYGRNDSRLT HTLNSTTHEY KIQGLSSLTT YTIDVAAVTA
VGTGLVTSST ISSGVPPDLP GAPSNLVISN ISPRSATLQF RPGYDGKTSI SRWIVEGQVG AIGDEEEWVT
LYEEENEPDA QMLEIPNLTP YTHYRFRMKQ VNIVGPSPYS PSSRVIQTLQ APPDVAPTSV TVRTASETSL
RLRWVPLPDS QYNGNPESVG YRIKYWRSDL QSSAVAQVVS DRLEREFTIE ELEEWMEYEL QMQAFNAVGA
GPWSEVVRGR TRESVPSAAP ENVSAEAVSS TQILLTWTSV PEQDQNGLIL GYKILFRAKD LDPEPRSHIV
RGNHTQSALL AGLRKFVLYE LQVLAFTRIG NGVPSTPLIL ERTKDDAPGP PVRLVFPEVR LTSVRIVWQP
PEEPNGIILG YQIAYRLASS SPHTFTTVEV GATVRQFTAT DLAPESAYIF RLSAKTRQGW GEPLEATVIT
TEKRERPAPP RELLVPQAEV TARSLRLQWV PGSDGASPIR YFTMQVRELP RGEWQTYSSS ISHEATACVV
DRLRPFTSYK LRLKATNDIG DSDFSSETEA VTTLQDVPGE PPGSVSATPH TTSSVLIQWQ PPRDESLNGL
LQGYRIYYRE LEYEAGSGTE AKTLKNPIAL HAELTAQSSF KTVNSSSTST MCELTHLKKY RRYEVIMTAY
NIIGESPASA PVEVFVGEAA PAMAPQNVQV TPLTASQLEV TWDPPPPESQ NGNIQGYKIY YWEADSQNET
EKMKVLFLPE PVVRLKNLTS HTKYLVSISA FNAAGDGPKS DPQQGRTHQA APGAPSFLAF SEITSTTLNV
SWGEPAAANG ILQGYRVVYE PLAPVQGVSK VVTVEVRGNW QRWLKVRDLT KGVTYFFRVQ ARTITYGPEL
QANITAGPAE GSPGSPRDVL VTKSASELTL QWTEGHSGDT PTTGYVIEAR PSDEGLWDMF VKDIPRSATS
YTLSLDKLRQ GVTYEFRVVA VNEAGYGEPS NPSTAVSAQV EAPFYEEWWF LLVMALSSLI VILLVVFALV
LHGQNKKYKN CSTGKGISTM EESVTLDNGG FAALELSSRH LNVKSTFSKK NGTRSPPRPS PGGLHYSDED
ICNKYNGAVL TESVSLKEKS ADASESEATD SDYEDALPKH SFVNHYMSDP TYYNSWKRRA QGRAPAPHRY
EAVAGSEAGA QLHPVITTQS AGGVYTPAGP GARTPLTGFS SFV

>IPI:IPI00292043.4 Homo sapiens (Human) ISOFORM 1 OF PROTEIN SIDEKICK-2 PRECURSOR. [MASS=239121]

MWGLLIWTLL ALHQIRAARA QDDVSPYFKT EPVRTQVHLE GNRLVLTCMA EGSWPLEFKW LHNNRELTKF
SLEYRYMITS LDRTHAGFYR CIVRNRMGAL LQRQTEVQVA DMGSFEEGEK HQSVSHGEAA VIRAPRIASF
PQPQVTWFRD GRKIPPSSRA ITLENTLVIL STVAPDAGRY YVQAVNDKNG DNKTSQPITL TVEVGGPADP
IAPTIIIPPK NTSVVAGTSE VTLECVANAR PLIKLHIIWK KDGVLLSGGI SDHNRRLTIP NPTGSDAGYY
ECEAVLRSSS VPSVVRGAYL SVLEPPQFVK EPERHITAEM EKVVDIPCQA KGVPPPSITW YKDAAVVEVE
KLTRFRQRND GGLQISGLVP DDTGMFQCFA RNAAGEVQTS TYLAVTSIAP NITRGPLDST VIDGMSVVLA
CETSGAPRPA ITWQKGERIL ASGSVQLPRF TPLESGSLLI SPTHISDAGT YTCLATNSRG VDEASADLVV
WARTRITKPP QDQSVIKGTQ ASMVCGVTHD PRVTIRYIWE KDGATLGTES HPRIRLDRNG SLHISQTWSG
DIGTYTCRVI SAGGNDSRSA HLRVRQLPHA PEHPVATLST VERRAINLTW TKPFDGNSPL IRYILEMSEN
NAPWTVLLAS VDPKATSVTV KGLVPARSYQ FRLCAVNDVG KGQFSKDTER VSLPEEPPTA PPQNVIASGR
TNQSIMIQWQ PPPESHQNGI LKGYIIRYCL AGLPVGYQFK NITDADVNNL LLEDLIIWTN YEIEVAAYNS
AGLGVYSSKV TEWTLQGVPT VPPGNVHAEA TNSTTIRFTW NAPSPQFING INQGYKLIAW EPEQEEEVTM
VTARPNFQDS IHVGFVSGLK KFTEYFTSVL CFTTPGDGPR STPQLVRTHE DVPGPVGHLS FSEILDTSLK
VSWQEPGEKN GILTGYRISW EEYNRTNTRV THYLPNVTLE YRVTGLTALT TYTIEVAAMT SKGQGQVSAS

TISSGVPPEL PGPPTNLGIS NIGPRSVTLQ FRPGYDGKTS ISRWLVEAQV GVVGEGEEWL LIHQLSNEPD
ARSMEVPDLN PFTCYSFRMR QVNIVGTSPP SQPSRKIQTL QAPPDMAPAN VSLRTASETS LWLRWMPLPE
MEYNGNPESV GYKIKYSRSD GHGKTLSHVV QDRVERDYTI EDLEEWTEYR VQVQAFNAIG SGPWSQTVVG
RTRESVPSSG PTNVSALATT SSSMLVRWSE VPEADRNGLV LGYKVMYKEK DSDTQPRFWL VEGNSSRSAQ
LTGLGKYVLY EVQVLAFTRI GDGSPSHPPI LERTLDDVPG PPMGILFPEV RTTSVRLIWQ PPAAPNGIIL
AYQITHRLNT TTANTATVEV LAPSARQYTA TGLKPESVYL FRITAQTRKG WGEAAEALVV TTEKRDRPQP
PSRPMVQQED VRARSVLLSW EPGSDGLSPV RYYTIQTREL PSGRWALHSA SVSHNASSFI VDRLKPFTSY
KFRVKATNDI GDSEFSEESE SLTTLQAAPD EAPTILSVTP HTTTSVLIRW QPPAEDKING ILLGFRIRYR
ELLYEGLRGF TLRGINNPGA TWAELTSMYS MRNLSRPSLT QYELDNLNKH RRYEIRMSVY NAVGEGPSSP
PQEVFVGEAV PTAAPRNVVV HGATATQLDV TWEPPPLDSQ NGDIQGYKIY FWEAQRGNLT ERVKTLFLAE
NSVKLKNLTG YTAYMVSVAA FNAAGDGPRS TPTQGQTQQA APSAPSSVKF SELTTTSVNV SWEAPQFPNG
ILEGYRLVYE PCSPVDGVSK IVTVDVKGNS PLWLKVKDLA EGVTYRFRIR AKTFTYGPEI EANVTTGPGE
GAPGPPGVPI IVRYSSAIAI HWSSGDPGKG PITRYVIEAR PSDEGLWDIL IKDIPKEVSS YTFSMDILKP
GVSYDFRVIA VNDYGFGTPS SPSQSVPAQK ANPFYEEWWF LVVIALVGLI FILLLVFVLI IRGQSKKYAK
KTDSGNSAKS GALGHSEMMS LDESSFPALE LNNRRLSVKN SFCRKNGLYT RSPPRPSPGS LHYSDEDVTK
YNDLIPAESS SLTEKPSEIS DSQGSDSEYE VDSNHQKAHS FVNHYISDPT YYNSWRRQQK GISRAQAYSY
TESDSGEPDH TTVTNSTSTQ QGSLFRPKAS RTPTPQNPPN PPSQQSTLYR PPSSLAPGSR APIAGFSSFV

Protein	Peptide	Cell Line	PSMs	Ratio(BACE/total)
CD276	SPTGAVEVQVPEDPVVALVGTDATLR	HEK	2	0.72
CD276	VADEGSFTCFVSIR	HEK	1	0.68

>IPI:IPI00410488.2 Homo sapiens (Human) ISOFORM 1 OF CD276 ANTIGEN PRECURSOR. [MASS=57235]

MLRRRGSPGM GVHVGAALGA LWFCLTGALE VQVPEDPVVA LVGTDATLCC SFSPEPGFSL AQLNLIWQLT
DTKQLVHSFA EGQDQGSAYA NRTALFPDLL AQGNASLRLQ RVRVADEGSF TCFVSIRDFG SAAVSLQVAA
PYSKPSMTLE PNKDLRPGDT VTITCSSYQG YPEAEVFWQD GQGVPLTGNV TTSQMANEQG LFDVHSILRV
VLGANGTYSC LVRNPVLQQD AHSSVTITPQ RSPTGAVEVQ VPEDPVVALV GTDATLRCSF SPEPGFSLAQ
LNLIWQLTDT KQLVHSFTEG RDQGSAYANR TALFPDLLAQ GNASLRLQRV RVADEGSFTC FVSIRDFGSA
AVSLQVAAPY SKPSMTLEPN KDLRPGDTVT ITCSSYRGYP EAEVFWQDGQ GVPLTGNVTT SQMANEQGLF
DVHSVLRVVL GANGTYSCLV RNPVLQQDAH GSVTITGQPM TFPPEALWVT VGLSVCLIAL LVALAFVCWR
KIKQSCEEEN AGAEDQDGEG EGSKTALQPL KHSDSKEDDG QEIA

Protein	Peptide	Cell Line	PSMs	Ratio(BACE/total)
LRP11	GSGGYSAMPDAIIR	HEK	6	0.96
LRP11	QLQQERPQEELELELR	HeLa	2	0.82
LRP11	FALHSGYSSYSLSR	HEK	1	0.95
LRP4	TVLIWENLDRPR	HEK	7	0.89

>IPI:IPI00045841.5 Homo sapiens (Human) ISOFORM 1 OF LOW-DENSITY LIPOPROTEIN RECEPTOR-RELATED PROTEIN 11 PRECURSOR. [MASS=53311]

MASVAQESAG SQRRLPPRHG ALRGLLLLCL WLPSGRAALP PAAPLSELHA QLSGVEQLLE EFRRQLQQER
PQEELELELR AGGGPQEDCP GPGSGGYSAM PDAIIRTKDS LAAGASFLRA PAAVRGWRQC VAACCSEPRC
SVAVVELPRR PAPPAAVLGC YLFNCTARGR NVCKFALHSG YSSYSLSRAP DGAALATARA SPRQEKDAPP
LSKAGQDVVL HLPTDGVVLD GRESTDDHAI VQYEWALLQG DPSVDMKVPQ SGTLKLSHLQ EGTYTFQLTV
TDTAGQRSSD NVSVTVLRAA YSTGGCLHTC SRYHFFCDDG CCIDITLACD GVQQCPDGSD EDFCQNLGLD
RKMVTHTAAS PALPRTTGPS EDAGGDSLVE KSQKATAPNK PPALSNTEKR NHSAFWGPES QIIPVMPDSS
SSGKNRKEES YIFESKGDGG GGEHPAPETG AVLPLALGLA ITALLLLMVA CRLRLVKQKL KKARPITSEE
SDYLINGMYL

>IPI:IPI00306851.3 Homo sapiens (Human) LOW-DENSITY LIPOPROTEIN RECEPTOR-RELATED PROTEIN 4 PRECURSOR. [MASS=215965]

MRRQWGALLL GALLCAHAVA LGLRAGERTR SGPGSSSPSG GISGGASAGS GLGRGAGLGR GAGLASSPEC
ACGRSHFTCA VSALGECTCI PAQWQCDGDN DCGDHSDEDG CILPTCSPLD FHCDNGKCIR RSWVCDGDND
CEDDSDEQDC PPRECEEDEF PCQNGYCIRS LWHCDGDNDC GDNSDEQCDM RKCSDKEFRC SDGSCIAEHW
YCDGDTDCKD GSDEENCPSA VPAPPCNLEE FQCAYGRCIL DIYHCDGDDD CGDWSDESDC SSHQPCRSGE
FMCDSGLCIN AGWRCDGDAD CDDQSDERNC TTSMCTAEQF RCHSGRCVRL SWRCDGEDDC ADNSDEENCE

NTGSPQCALD QFLCWNGRCI GQRKLCNGVN DCGDNSDESP QQNCRPRTGE ENCNVNNGGC AQKCQMVRGA VQCTCHTGYR LTEDGHTCQD VNECAEEGYC SQGCTNSEGA FQCWCETGYE LRPDRRSCKA LGPEPVLLFA NRIDIRQVLP HRSEYTLLLN NLENAIALDF HHRRELVFWS DVTLDRILRA NLNGSNVEEV VSTGLESPGG LAVDWVHDKL YWTDSGTSRI EVANLDGAHR KVLLWQNLEK PRAIALHPME GTIYWTDWGN TPRIEASSMD GSGRRIIADT HLFWPNGLTI DYAGRRMYWV DAKHHVIERA NLDGSHRKAV ISQGLPHPFA ITVFEDSLYW TDWHTKSINS ANKFTGKNQE IIRNKLHFPM DIHTLHPQRQ PAGKNRCGDN NGGCTHLCLP SGQNYTCACP TGFRKISSHA CAQSLDKFLL FARRMDIRRI SFDTEDLSDD VIPLADVRSA VALDWDSRDD HVYWTDVSTD TISRAKWDGT GQEVVVDTSL ESPAGLAIDW VTNKLYWTDA GTDRIEVANT DGSMRTVLIW ENLDRPRDIV VEPMGGYMYW TDWGASPKIE RAGMDASGRQ VIISSNLTWP NGLAIDYGSQ RLYWADAGMK TIEFAGLDGS KRKVLIGSQL PHPFGLTLYG ERIYWTDWQT KSIQSADRLT GLDRETLQEN LENLMDIHVF HRRRPPVSTP CAMENGGCSH LCLRSPNPSG FSCTCPTGIN LLSDGKTCSP GMNSFLIFAR RIDIRMVSLD IPYFADVVVP INITMKNTIA IGVDPQEGKV YWSDSTLHRI SRANLDGSQH EDIITTGLQT TDGLAVDAIG RKVYWTDTGT NRIEVGNLDG SMRKVLVWQN LDSPRAIVLY HEMGFMYWTD WGENAKLERS GMDGSDRAVL INNNLGWPNG LTVDKASSQL LWADAHTERI EAADLNGANR HTLVSPVQHP YGLTLLDSYI YWTDWQTRSI HRADKGTGSN VILVRSNLPG LMDMQAVDRA QPLGFNKCGS RNGGCSHLCL PRPSGFSCAC PTGIQLKGDG KTCDPSPETY LLFSSRGSIR RISLDTSDHT DVHVPVPELN NVISLDYDSV DGKVYYTDVF LDVIRRADLN GSNMETVIGR GLKTTDGLAV DWVARNLYWT DTGRNTIEAS RLDGSCRKVL INNSLDEPRA IAVFPRKGYL FWTDWGHIAK IERANLDGSE RKVLINTDLG WPNGLTLDYD TRRIYWVDAH LDRIESADLN GKLRQVLVSH VSHPFALTQQ DRWIYWTDWQ TKSIQRVDKY SGRNKETVLA NVEGLMDIIV VSPQRQTGTN ACGVNNGGCT HLCFARASDF VCACPDEPDS RPCSLVPGLV PPAPRATGMS EKSPVLPNTP PTTLYSSTTR TRTSLEEVEG RCSERDARLG LCARSNDAVP AAPGEGLHIS YAIGGLLSIL LILVVIAALM LYRHKKSKFT DPGMGNLTYS NPSYRTSTQE VKIEAIPKPA MYNQLCYKKE GGPDHNYTKE KIKIVEGICL LSGDDAEWDD LKQLRSSRGG LLRDHVCMKT DTVSIQASSG SLDDTETEQL LQEEQSECSS VHTAAPTPERR GSLPDTGWKH ERKLSSESQV

Protein	Peptide	Cell Line	PSMs	Ratio(BACE/total)
PAM	KAGIEVQEIKEAEAVVETK	HEK	11	0.77
PAM	FTLTEKLEHR	HEK	5	0.73
PAM	GDHVWDGNSFDSK	HEK	3	0.71

>IPI:IPI00177543.5 Homo sapiens (Human) ISOFORM 1 OF PEPTIDYL-GLYCINE ALPHA-AMIDATING MONOOXYGENASE PRECURSOR. [MASS=108516]

MAGRVPSLLV LLVFPSSCLA FRSPLSVFKR FKETTRPFSN ECLGTTRPVV PIDSSDFALD IRMPGVTPKQ SDTYFCMSMR IPVDEEAFVI DFKPRASMDT VHHMLLFGCN MPSSTGSYWF CDEGTCTDKA NILYAWARNA PPTRLPKGVG FRVGGETGSK YFVLQVHYGD ISAFRDNNKD CSGVSLHLTR LPQPLIAGMY LMMSVDTVIP AGEKVVNSDI SCHYKNYPMH VFAYRVHTHH LGKVVSGYRV RNGQWTLIGR QSPQLPQAFY PVGHPVDVSF GDLLAARCVF TGEGRTEATH IGGTSSDEMC NLYIMYYMEA KHAVSFMTCT QNVAPDMFRT IPPEANIPIP VKSDMVMMHE HHKETEYKDK IPLLQQPKRE EEEVLDQGDF YSLLSKLLGE REDVVHVHKY NPTEKAESES DLVAEIANVV QKKDLGRSDA REGAEHERGN AILVRDRIHK FHRLVSTLRP PESRVFSLQQ PPPGEGTWEP EHTGDFHMEE ALDWPGVYLL PGQVSGVALD PKNNLVIFHR GDHVWDGNSF DSKFVYQQIG LGPIEEDTIL VIDPNNAAVL QSSGKNLFYL PHGLSIDKDG NYWVTDVALH QVFKLDPNNK EGPVLILGRS MQPGSDQNHF CQPTDVAVDP GTGAIYVSDG YCNSRIVQFS PSGKFITQWG EESSGSSPLP GQFTVPHSLA LVPLLGQLCV ADRENGRIQC FKTDTKEFVR EIKHSSFGRN VFAISYIPGL LFAVNGKPHF GDQEPVQGFV MNFSNGEIID IFKPVRKHFD MPHDIVASED GTVYIGDAHT NTVWKFTLTE KLEHRSVKKA GIEVQEIKEA EAVVETKMEN KPTSSELQKM QEKQKLIKEP GSGVPVVLIT TLLVIPVVVL LAIAIFIRWK KSRAFGGKNS EHKLETSSGR VLGRFRGKGS GGLNLGNFFA SRKGYSRKGF DRLSTEGSDQ EKEDDGSESE EEYSAPLPAL APSSS

Protein	Peptide	Cell Line	PSMs	Ratio(BACE/total)
SDC4	ESIRETEVIDPQDLLEGR	HEK	5	0.96
SDC4	RISPVEESEDVSN	HEK	2	0.97
SDC4	ETEVIDPQDLLEGR	HEK	2	0.92
SDC4	RISPVEESEDVSNKVSM	HEK	1	1.00

>IPI:IPI00011564.1 Homo sapiens (Human) SYNDECAN-4 PRECURSOR. [MASS=21642]

MAPARLFALL LFFVGGVAES IRETEVIDPQ DLLEGRYFSG ALPDDEDVVG PGQESDDFEL SGSGDLDDLE DSMIGPEVVH PLVPLDNHIP ERAGSGSQVP TEPKKLEENE VIPKRISPVE ESEDVSNKVS MSSTVQGSNI FERTEVLAAL IVGGIVGILF AVFLILLLMY RMKKKDEGSY DLGKKPIYKK APTNEFYA

Protein	Peptide	Cell Line	PSMs	Ratio(BACE/total)
PODXL2	ATFNPAQDKCGIR	HEK	1	0.97
PODXL2	LASVPGSQTVVVK	HEK	1	0.92

>IPI:IPI00299116.1 Homo sapiens (Human) PODOCALYXIN-LIKE PROTEIN 1 PRECURSOR. [MASS=55596]

**MRCALALSAL LLLLSTPPLL PSSPSPSPSP SPSQNATQTT TDSSNKTAPT PASSVTIMAT DTAQQSTVPT
SKANEILASV KATTLGVSSD SPGTTTLAQQ VSGPVNTTVA RGGGSGNPTT TIESPKSTKS ADTTTVATST
ATAKPNTTSS QNGAEDTTNS GGKSSHSVTT DLTSTKAEHL TTPHPTSPLS PRQPTLTHPV ATPTSSGHDH
LMKISSSSST VAIPGYTFTS PGMTTTLPSS VISQRTQQTS SQMPASSTAP SSQETVQPTS PATALRTPTL
PETMSSSPTA ASTTHRYPKT PSPTVAHESN WAKCEDLETQ TQSEKQLVLN LTGNTLCAGG ASDEKLISLI
CRAVKATFNP AQDKCGIRLA SVPGSQTVVV KEITIHTKLP AKDVYERLKD KWDELKEAGV SDMKLGDQGP
PEEAEDRFSM PLIITIVCMA SFLLLVAALY GCCHQRLSQR KDQQRLTEEL QTVENGYHDN PTLEVMETSS
EMQEKKVVSL NGELGDSWIV PLDNLTKDDL DEEEDTHL**

Protein	Peptide	Cell Line	PSMs	Ratio(BACE/total)
LRIG1/2/3	DGGTDFPAAR	HEK	1	0.92
LRIG1	VLDLDHNEISGTIEDTSGAFSGLDSLSK	HEK	1	0.77
LRIG3	LECAAVGHPAPQIAWQK	HEK	1	1.00

>IPI:IPI00000775.2 Homo sapiens (Human) ISOFORM 1 OF LEUCINE-RICH REPEATS AND IMMUNOGLOBULIN-LIKE DOMAINS PROTEIN 1 PRECURSOR. [MASS=119053]

**MARPVRGGLG APRRSPCLLL LWLVLVRLEP VTAAAGPRAP CAAACTCAGD SLDCGGRGLA ALPGDLPSWT
RSLNLSYNKL SEIDPAGFED LPNLQEVYLN NNELTAVPSL GAASSHVVSL FLQHNKIRSV EGSQLKAYLS
LEVLDLSLNN ITEVRNTCFP HGPPIKELNL AGNRIGTLEL GAFDGLSRSL LTLRLSKNRI TQLPVRAFKL
PRLTQLDLNR NRIRLIEGLT FQGLNSLEVL KLQRNNISKL TDGAFWGLSK MHVLHLEYNS LVEVNSGSLY
GLTALHQLHL SNNSIARIHR KGWSFCQKLH ELVLSFNNLT RLDEESLAEL SSLSVLRLSH NSISHIAEGA
FKGLRSLRVL DLDHNEISGT IEDTSGAFSG LDSLSKLTLF GNKIKSVAKR AFSGLEGLEH LNLGGNAIRS
VQFDAFVKMK NLKELHISSD SFLCDCQLKW LPPWLIGRML QAFVTATCAH PESLKGQSIF SVPPESFVCD
DFLKPQIITQ PETTMAMVGK DIRFTCSAAS SSSSPMTFAW KKDNEVLTNA DMENFVHVHA QDGEVMEYTT
ILHLRQVTFG HEGRYQCVIT NHFGSTYSHK ARLTVNVLPS FTKTPHDITI RTTTVARLEC AATGHPNPQI
AWQKDGGTDF PAARERRMHV MPDDDVFFIT DVKIDDAGVY SCTAQNSAGS ISANATLTVL ETPSLVVPLE
DRVVSVGETV ALQCKATGNP PPRITWFKGD RPLSLTERHH LTPDNQLLVV QNVVAEDAGR YTCEMSNTLG
TERAHSQLSV LPAAGCRKDG TTVGIFTIAV VSSIVLTSLV WVCIIYQTRK KSEEYSVTNT DETVVPPDVP
SYLSSQGTLS DRQETVVRTE GGPQANGHIE SNGVCPRDAS HFPEPDTHSV ACRQPKLCAG SAYHKEPWKA
MEKAEGTPGP HKMEHGGRVV CSDCNTEVDC YSRGQAFHPQ PVSRDSAQPS APNGPEPGGS DQEHSPHHQC
SRTAAGSCPE CQGSLYPSNH DRMLTAVKKK PMASLDGKGD SSWTLARLYH PDSTELQPAS SLTSGSPERA
EAQYLLVSNG HLPKACDASP ESTPLTGQLP GKQRVPLLLA PKS**

>IPI:IPI00184265.2 Homo sapiens (Human) ISOFORM 1 OF LEUCINE-RICH REPEATS AND IMMUNOGLOBULIN-LIKE DOMAINS PROTEIN 3 PRECURSOR. [MASS=123434]

**MSAPSLRARA AGLGLLLCAV LGRAGRSDSG GRGELGQPSG VAAERPCPTT CRCLGDLLDC SRKRLARLPE
PLPSWVARLD LSHNRLSFIK ASSMSHLQSL REVKLNNNEL ETIPNLGPVS ANITLLSLAG NRIVEILPEH
LKEFQSLETL DLSSNNISEL QTAFPALQLK YLYLNSNRVT SMEPGYFDNL ANTLLVLKLN RNRISAIPPK
MFKLPQLQHL ELNRNKIKNV DGLTFQGLGA LKSLKMQRNG VTKLMDGAFW GLSNMEILQL DHNNLTEITK
GWLYGLLMLQ ELHLSQNAIN RISPDAWEFC QKLSELDLTF NHLSRLDDSS FLGLSLLNTL HIGNNRVSYI
ADCAFRGLSS LKTLDLKNNE ISWTIEDMNG AFSGLDKLRR LILQGNRIRS ITKKAFTGLD ALEHLDLSDN
AIMSLQGNAF SQMKKLQQLH LNTSSLLCDC QLKWLPQWVA ENNFQSFVNA SCAHPQLLKG RSIFAVSPDG
FVCDDFPKPQ ITVQPETQSA IKGSNLSFIC SAASSSDSPM TFAWKKDNEL LHDAEMENYA HLRAQGGEVM
EYTTILRLRE VEFASEGKYQ CVISNHFGSS YSVKAKLTVN MLPSFTKTPM DLTIRAGAMA RLECAAVGHP
APQIAWQKDG GTDFPAARER RMHVMPEDDV FFIVDVKIED IGVYSCTAQN SAGSISANAT LTVLETPSFL
RPLLDRTVTK GETAVLQCIA GGSPPPKLNW TKDDSPLVVT ERHFFAAGNQ LLIIVDSDVS DAGKYTCEMS
NTLGTERGNV RLSVIPTPTC DSPQMTAPSL DDDGWATVGV VIIAVVCCVV GTSLVWVVII YHTRRRNEDC
SITNTDETNL PADIPSYLSS QGTLADRQDG YVSSESGSHH QFVTSSGAGF FLPQHDSSGT CHIDNSSEAD
VEAATDLFLC PFLGSTGPMY LKGNVYGSDP FETYHTGCSP DPRTVLMDHY EPSYIKKKEC YPCSHPSEES**

CERSFSNISW PSHVRKLLNT SYSHNEGPGM KNLCLNKSSL DFSANPEPAS VASSNSFMGT FGKALRRPHL
DAYSSFGQPS DCQPRAFYLK AHSSPDLDSG SEEDGKERTD FQEENHICTF KQTLENYRTP NFQSYDLDT

Protein	Peptide	Cell Line	PSMs	Ratio(BACE/total)
NCAM1	FIIEYEDAMHKPGLWHHQTEVSGTQTTAQLK	HEK	5	0.84
NCAM1 N(	KVDKNDEAEYICIAENK	HEK	4	0.93
NCAM1 N(	DGEQIEQEEDDEKYIFSDDSSQLTIK	HEK	1	0.98
NCAM1 14	TQPVQGEPSAPK	HEK	1	0.79
NCAML1	VGEEDDGEYRCLAENSLGSAR	HeLa	5	0.73
NCAML1	LGTAMSHEIR	HeLa	2	0.84

>IPI:IPI00333776.4 Homo sapiens (Human) ISOFORM 1 OF NEURONAL CELL ADHESION MOLECULE PRECURSOR. [MASS=144074]

**MQLKIMPKKK RLSAGRVPLI LFLCQMISAL EVPLDPKLLE DLVQPPTITQ QSPKDYIIDP RENIVIQCEA
KGKPPPSFSW TRNGTHFDID KDPLVTMKPG TGTLIINIMS EGKAETYEGV YQCTARNERG AAVSNNIVVR
PSRSPLWTKE KLEPITLQSG QSLVLPCRPP IGLPPPIIFW MDNSFQRLPQ SERVSQGLNG DLYFSNVLPE
DTREDYICYA RFNHTQTIQQ KQPISVKVIS VDELNDTIAA NLSDTEFYGA KSSRERPPTF LTPEGNASNK
EELRGNVLSL ECIAEGLPTP IIYWAKEDGM LPKNRTVYKN FEKTLQIIHV SEADSGNYQC IAKNALGAIH
HTISVRVKAA PYWITAPQNL VLSPGEDGTL ICRANGNPKP RISWLTNGVP IEIAPDDPSR KIDGDTIIFS
NVQERSSAVY QCNASNEYGY LLANAFVNVL AEPPRILTPA NTLYQVIANR PALLDCAFFG SPLPTIEWFK
GAKGSALHED IYVLHENGTL EIPVAQKDST GTYTCVARNK LGMAKNEVHL EIKDPTWIVK QPEYAVVQRG
SMVSFECKVK HDHTLSLTVL WLKDNRELPS DERFTVDKDH LVVADVSDDD SGTYTCVANT TLDSVSASAV
LSVVAPTPTP APVYDVPNPP FDLELTDQLD KSVQLSWTPG DDNNSPITKF IIEYEDAMHK PGLWHHQTEV
SGTQTTAQLK LSPYVNYSFR VMAVNSIGKS LPSEASEQYL TKASEPDKNP TAVEGLGSEP DNLVITWKPL
NGFESNGPGL QYKVSWRQKD GDDEWTSVVV ANVSKYIVSG TPTFVPYLIK VQALNDMGFA PEPAVVMGHS
GEDLPMVAPG NVRVNVVNST LAEVHWDPVP LKSIRGHLQG YRAIIYYWKT QSSSKRNRRH IEKKILTFQG
SKTHGMLPGL EPFSHYTLNV RVVNGKGEGP ASPDRVFNTP EGVPSAPSSL KIVNPTLDSL TLEWDPPSHP
NGILTEYTLK YQPINSTHEL GPLVDLKIPA NKTRWTLKNL NFSTRYKFYF YAQTSAGSGS QITEEAVTTV
DEAGILPPDV GAGKVQAVNP RISNLTAAAA ETYANISWEY EGPEHVNFYV EYGVAGSKEE WRKEIVNGSR
SFFGLKGLMP GTAYKVRVGA VGDSGFVSSE DVFETGPAMA SRQVDIATQG WFIGLMCAVA LLILILLIVC
FIRRNKGGKY PVKEKEDAHA DPEIQPMKED DGTFGEYSDA EDHKPLKKGS RTPSDRTVKK EDSDDSLVDY
GEGVNGQFNE DGSFIGQYSG KKEKEPAEGN ESSEAPSPVN AMNSFV**

>IPI:IPI00435020.3 Homo sapiens (Human) NEURAL CELL ADHESION MOLECULE 1, 140 KDA ISOFORM PRECURSOR. [MASS=93361]

**MLQTKDLIWT LFFLGTAVSL QVDIVPSQGE ISVGESKFFL CQVAGDAKDK DISWFSPNGE KLTPNQQRIS
VVWNDDSSST LTIYNANIDD AGIYKCVVTG EDGSESEATV NVKIFQKLMF KNAPTPQEFR EGEDAVIVCD
VVSSLPPTII WKHKGRDVIL KKDVRFIVLS NNYLQIRGIK KTDEGTYRCE GRILARGEIN FKDIQVIVNV
PPTIQARQNI VNATANLGQS VTLVCDAEGF PEPTMSWTKD GEQIEQEEDD EKYIFSDDSS QLTIKKVDKN
DEAEYICIAE NKAGEQDATI HLKVFAKPKI TYVENQTAME LEEQVTLTCE ASGDPIPSIT WRTSTRNISS
EEKTLDGHMV VRSHARVSSL TLKSIQYTDA GEYICTASNT IGQDSQSMYL EVQYAPKLQG PVAVYTWEGN
QVNITCEVFA YPSATISWFR DGQLLPSSNY SNIKIYNTPS ASYLEVTPDS ENDFGNYNCT AVNRIGQESL
EFILVQADTP SSPSIDQVEP YSSTAQVQFD EPEATGGVPI LKYKAEWRAV GEEVWHSKWY DAKEASMEGI
VTIVGLKPET TYAVRLAALN GKGLGEISAA SEFKTQPVQG EPSAPKLEGQ MGEDGNSIKV NLIKQDDGGS
PIRHYLVRYR ALSSEWKPEI RLPSGSDHVM LKSLDWNAEY EVYVVAENQQ GKSKAAHFVF RTSAQPTAIP
ANGSPTSGLS TGAIVGILIV IFVLLLVVVD ITCYFLNKCG LFMCIAVNLC GKAGPGAKGK DMEEGKAAFS
KDESKEPIVE VRTEEERTPN HDGGKHTEPN ETTPLTEPEK GPVEAKPECQ ETETKPAPAE VKTVPNDATQ
TKENESKA**

>IPI:IPI00027087.1 Homo sapiens (Human) ISOFORM 1 OF NEURAL CELL ADHESION MOLECULE L1 PRECURSOR. [MASS=140003]

**MVVALRYVWP LLLCSPCLLI QIPEEYEGHH VMEPPVITEQ SPRRLVVFPT DDISLKCEAS GKPEVQFRWT
RDGVHFKPKE ELGVTVYQSP HSGSFTITGN NSNFAQRFQG IYRCFASNKL GTAMSHEIRL MAEGAPKWPK
ETVKPVEVEE GESVVLPCNP PPSAEPLRIY WMNSKILHIK QDERVTMGQN GNLYFANVLT SDNHSDYICH
AHFPGTRTII QKEPIDLRVK ATNSMIDRKP RLLFPTNSSS HLVALQGQPL VLECIAEGFP TPTIKWLRPS
GPMPADRVTY QNHNKTLQLL KVGEEDDGEY RCLAENSLGS ARHAYYVTVE AAPYWLHKPQ SHLYGPGETA**

RLDCQVQGRP QPEVTWRING IPVEELAKDQ KYRIQRGALI LSNVQPSDTM VTQCEARNRH GLLLANAYIY
VVQLPAKILT ADNQTYMAVQ GSTAYLLCKA FGAPVPSVQW LDEDGTTVLQ DERFFPYANG TLGIRDLQAN
DTGRYFCLAA NDQNNVTIMA NLKVKDATQI TQGPRSTIEK KGSRVTFTCQ ASFDPSLQPS ITWRGDGRDL
QELGDSDKYF IEDGRLVIHS LDYSDQGNYS CVASTELDVV ESRAQLLVVG SPGPVPRLVL SDLHLLTQSQ
VRVSWSPAED HNAPIEKYDI EFEDKEMAPE KWYSLGKVPG NQTSTTLKLS PYVHYTFRVT AINKYGPGEP
SPVSETVVTP EAAPEKNPVD VKGEGNETTN MVITWKPLRW MDWNAPQVQY RVQWRPQGTR GPWQEQIVSD
PFLVVSNTST FVPYEIKVQA VNSQGKGPEP QVTIGYSGED YPQAIPELEG IEILNSSAVL VKWRPVDLAQ
VKGHLRGYNV TYWREGSQRK HSKRHIHKDH VVVPANTTSV ILSGLRPYSS YHLEVQAFNG RGSGPASEFT
FSTPEGVPGH PEALHLECQS NTSLLLRWQP PLSHNGVLTG YVLSYHPLDE GGKGQLSFNL RDPELRTHNL
TDLSPHLRYR FQLQATTKEG PGEAIVREGG TMALSGISDF GNISATAGEN YSVVSWVPKE GQCNFRFHIL
FKALGEEKGG ASLSPQYVSY NQSSYTQWDL QPDTDYEIHL FKERMFRHQM AVKTNGTGRV RLPPAGFATE
GWFIGFVSAI ILLLLVLLIL CFIKRSKGGK YSVKDKEDTQ VDSEARPMKD ETFGEYRSLE SDNEEKAFGS
SQPSLNGDIK PLGSDDSLAD YGGSVDVQFN EDGSFIGQYS GKKEKEAAGG NDSSGATSPI NPAVALE

Protein	Peptide	Cell Line	PSMs	Ratio(BACE/total)
SEMA4B	ETQQWYTVTHPVPTPRPGACITNSAR	HEK	6	0.83
SEMA4B	DTLFYGVFTSQWHR	HeLa	1	0.75
SEMA4C	AEVWWNLVPR	HEK	7	0.88
SEMA4C	HAGAYHCFSEEQGAR	HEK	1	0.96
SEMA6A	PGCCAGSSSLER	HEK	1	0.70
SEMA6D	TSIDFPDETLSFIK	HEK	1	0.92

>IPI:IPI00419724.2 Homo sapiens (Human) SEMAPHORIN 4B PRECURSOR. [MASS=92766]
MLRTAMGLRS WLAAPWGALP PRPPLLLLLL LLLLLQPPPP TWALSPRISL PLGSEERPFL RFEAEHISNY
TALLLSRDGR TLYVGAREAL FALSSNLSFL PGGEYQELLW GADAEKKQQC SFKGKDPQRD CQNYIKILLP
LSGSHLFTCG TAAFSPMCTY INMENFTLAR DEKGNVLLED GKGRCPFDPN FKSTALVVDG ELYTGTVSSF
QGNDPAISRS QSLRPTKTES SLNWLQDPAF VASAYIPESL GSLQGDDDKI YFFFSETGQE FEFFENTIVS
RIARICKGDE GGERVLQQRW TSFLKAQLLC SRPDDGFPFN VLQDVFTLSP SPQDWRDTLF YGVFTSQWHR
GTTEGSAVCV FTMKDVQRVF SGLYKEVNRE TQQWYTVTHP VPTPRPGACI TNSARERKIN SSLQLPDRVL
NFLKDHFLMD GQVRSRMLLL QPQARYQRVA VHRVPGLHHT YDVLFLGTGD GRLHKAVSVG PRVHIIEELQ
IFSSGQPVQN LLLDTHRGLL YAASHSGVVQ VPMANCSLYR SCGDCLLARD PYCAWSGSSC KHVSLYQPQL
ATRPWIQDIE GASAKDLCSA SSVVSPSFVP TGEKPCEQVQ FQPNTVNTLA CPLLSNLATR LWLRNGAPVN
ASASCHVLPT GDLLLVGTQQ LGEFQCWSLE EGFQQLVASY CPEVVEDGVA DQTDEGGSVP VIISTSRVSA
PAGGKASWGA DRSYWKEFLV MCTLFVLAVL LPVLFLLYRH RNSMKVFLKQ GECASVHPKT CPVVLPPETR
PLNGLGPPST PLDHRGYQSL SDSPPGSRVF TESEKRPLSI QDSFVEVSPV CPRPRVRLGS EIRDSVV

>IPI:IPI00073763.4 Homo sapiens (Human) SEMAPHORIN-4C PRECURSOR. [MASS=92623]
MAPHWAVWLL AARLWGLGIG AEVWWNLVPR KTVSSGELAT VVRRFSQTGI QDFLTLTLTE PTGLLYVGAR
EALFAFSMEA LELQGAISWE APVEKKTECI QKGKNNQTEC FNFIRFLQPY NASHLYVCGT YAFQPKCTYV
NMLTFTLEHG EFEDGKGKCP YDPAKGHAGL LVDGELYSAT LNNFLGTEPI ILRNMGPHHS MKTEYLAFWL
NEPHFVGSAY VPESVGSFTG DDDKVYFFFR ERAVESDCYA EQVVARVARV CKGDMGGART LQRKWTTFLK
ARLACSAPNW QLYFNQLQAM HTLQDTSWHN TTFFGVFQAQ WGDMYLSAIC EYQLEEIQRV FEGPYKEYHE
EAQKWDRYTD PVPSPRPGSC INNWHRRHGY TSSLELPDNI LNFVKKHPLM EEQVGPRWSR PLLVKKGTNF
THLVADRVTG LDGATYTVLF IGTGDGWLLK AVSLGPWVHL IEELQLFDQE PMRSLVLSQS KKLLFAGSRS
QLVQLPVADC MKYRSCADCV LARDPYCAWS VNTSRCVAVG GHSGSLLIQH VMTSDTSGIC NLRGSKKVRP
TPKNITVVAG TDLVLPCHLS SNLAHARWTF GGRDLPAEQP GSFLYDARLQ ALVVMAAQPR HAGAYHCFSE
EQGARLAAEG YLVAVVAGPS VTLEARAPLE NLGLVWLAVV ALGAVCLVLL LLVLSLRRRL REELEKGAKA
TERTLVYPLE LPKEPTSPPF RPCPEPDEKL WDPVGYYYSD GSLKIVPGHA RCQPGGGPPS PPPGIPGQPL
PSPTRLHLGG GRNSNANGYV RLQLGGEDRG GLGHPLPELA DELRRKLQQR QPLPDSNPEE SSV

>IPI:IPI00002211.3 Homo sapiens (Human) ISOFORM 2 OF SEMAPHORIN-6A PRECURSOR. [MASS=116310]
MRSEALLLYF TLLHFAGAGF PEDSEPISIS HGNYTKQYPV FVGHKPGRNT TQRHRLDIQM IMIMNGTLYI
AARDHIYTVD IDTSHTEEIY CSKKLTWKSR QADVDTCRMK GKHKDECHNF IKVLLKKNDD ALFVCGTNAF
NPSCRNYKMD TLEPFGDEFS GMARCPYDAK HANVALFADG KLYSATVTDF LAIDAVIYRS LGESPTLRTV

KHDSKWLKEP YFVQAVDYGD YIYFFFREIA VEYNTMGKVV FPRVAQVCKN DMGGSQRVLE KQWTSFLKAR
LNCSVPGDSH FYFNILQAVT DVIRINGRDV VLATFSTPYN SIPGSAVCAY DMLDIASVFT GRFKEQKSPD
STWTPVPDER VPKPRPGCCA GSSSLERYAT SNEFPDDTLN FIKTHPLMDE AVPSIFNRPW FLRTMVRYRL
TKIAVDTAAG PYQNHTVVFL GSEKGIILKF LARIGNSGFL NDSLFLEEMS VYNSEKCSYD GVEDKRIMGM
QLDRASSSLY VAFSTCVIKV PLGRCERHGK CKKTCIASRD PYCGWIKEGG ACSHLSPNSR LTFEQDIERG
NTDGLGDCHN SFVALNDIST PLPDNEMSYN TVYGHSSSLL PSTTTSDSTA QEGYESRGGM LDWKHLLDSP
DSTDPLGAVS SHNHQDKKGV IRESYLKGHD QLVPVTLLAI AVILAFVMGA VFSGITVYCV CDHRRKDVAV
VQRKEKELTH SRRGSMSSVT KLSGLFGDTQ SKDPKPEAIL TPLMHNGKLA TPGNTAKMLI KADQHHLDLT
ALPTPESTPT LQQKRKPSRG SREWERNQNL INACTKDMPP MGSPVIPTDL PLRASPSHIP SVVVLPITQQ
GYQHEYVDQP KMSEVAQMAL EDQAATLEYK TIKEHLSSKS PNHGVNLVEN LDSLPPKVPQ REASLGPPGA
SLSQTGLSKR LEMHHSSSYG VDYKRSYPTN SLTRSHQATT LKRNNTNSSN SSHLSRNQSF GRGDNPPPAP
QRVDSIQVHS SQPSGQAVTV SRQPSLNAYN SLTRSGLKRT PSLKPDVPPK PSFAPLSTSM KPNDACT

>IPI:IPI00170551.1 Homo sapiens (Human) ISOFORM 2 OF SEMAPHORIN-6D PRECURSOR. [MASS=111730]

**MRVFLLCAYI LLLMVSQLRA VSFPEDDEPL NTVDYHYSRQ YPVFRGRPSG NESQHRLDFQ LMLKIRDTLY
IAGRDQVYTV NLNEMPKTEV IPNKKLTWRS RQQDRENCAM KGKHKDECHN FIKVFVPRND EMVFVCGTNA
FNPMCRYYRL STLEYDGEEI SGLARCPFDA RQTNVALFAD GKLYSATVAD FLASDAVIYR SMGDGSALRT
IKYDSKWIKE PHFLHAIEYG NYVYFFFREI AVEHNNLGKA VYSRVARICK NDMGGSQRVL EKHWTSFLKA
RLNCSVPGDS FFYFDVLQSI TDIIQINGIP TVVGVFTTQL NSIPGSAVCA FSMDDIEKVF KGRFKEQKTP
DSVWTAVPED KVPKPRPGCC AKHGLAEAYK TSIDFPDETL SFIKSHPLMD SAVPPIADEP WFTKTRVRYR
LTAISVDHSA GPYQNYTVIF VGSEAGMVLK VLAKTSPFSL NDSVLLEEIE AYNHAKCSAE NEEDKKVISL
QLDKDHHALY VAFSSCIIRI PLSRCERYGS CKKSCIASRD PYCGWLSQGS CGRVTPGMLA EGYEQDTEFG
NTAHLGDCHG VRWEVQSGES NQMVHMNVLI TCVFAAFVLG AFIAGVAVYC YRDMFVRKNR KIHKDAESAQ
SCTDSSGSFA KLNGLFDSPV KEYQQNIDSP KLYSNLLTSR KELPPNGDTK SMVMDHRGQP PELAALPTPE
STPVLHQKTL QAMKSHSEKA HGHGASRKET PQFFPSSPPP HSPLSHGHIP SAIVLPNATH DYNTSFSNSN
AHKAEKKLQN IDHPLTKSSS KRDHRRSVDS RNTLNDLLKH LNDPNSNPKA IMGDIQMAHQ NLMLDPMGSM
SEVPPKVPNR EASLYSPPST LPRNSPTKRV DVPTTPGVPM TSLERQRGYH KNSSQRHSIS AMPKNLNSPN
GVLLSRQPSM NRGGYMPTPT GAKVDYIQGT PVSVHLQPSL SRQSSYTSNG TLPRTGLKRT PSLKPDVPPK
PSFVPQTPSV RPLNKYTY**

Protein	Peptide	Cell Line	PSMs	Ratio(BACE/total)
SORL1	SSDVYVSYDYGK	HEK	2	0.71
SORT1	NCEEKDYTIWLAHSTDPEDYEDGCILGYK	HEK	8	0.84

>IPI:IPI00022608.1 Homo sapiens (Human) SORTILIN-RELATED RECEPTOR PRECURSOR. [MASS=248441]

**MATRSSRRES RLPFLFTLVA LLPPGALCEV WTQRLHGGSA PLPQDRGFLV VQGDPRELRL WARGDARGAS
RADEKPLRRK RSAALQPEPI KVYGQVSLND SHNQMVVHWA GEKSNVIVAL ARDSLALARP KSSDVYVSYD
YGKSFKKISD KLNFGLGNRS EAVIAQFYHS PADNKRYIFA DAYAQYLWIT FDFCNTLQGF SIPFRAADLL
LHSKASNLLL GFDRSHPNKQ LWKSDDFGQT WIMIQEHVKS FSWGIDPYDK PNTIYIERHE PSGYSTVFRS
TDFFQSRENQ EVILEEVRDF QLRDKYMFAT KVVHLLGSEQ QSSVQLWVSF GRKPMRAAQF VTRHPINEYY
IADASEDQVF VCVSHSNNRT NLYISEAEGL KFSLSLENVL YYSPGGAGSD TLVRYFANEP FADFHRVEGL
QGVYIATLIN GSMNEENMRS VITFDKGGTW EFLQAPAFTG YGEKINCELS QGCSLHLAQR LSQLLNLQLR
RMPILSKESA PGLIIATGSV GKNLASKTNV YISSSAGARW REALPGPHYY TWGDHGGIIT AIAQGMETNE
LKYSTNEGET WKTFIFSEKP VFVYGLLTEP GEKSTVFTIF GSNKENVHSW LILQVNATDA LGVPCTENDY
KLWSPSDERG NECLLGHKTV FKRRTPHATC FNGEDFDRPV VVSNCSCTRE DYECDFGFKM SEDLSLEVCV
PDPEFSGKSY SPPVPCPVGS TYRRTRGYRK ISGDTCSGGD VEARLEGELV PCPLAEENEF ILYAVRKSIY
RYDLASGATE QLPLTGLRAA VALDFDYEHN CLYWSDLALD VIQRLCLNGS TGQEVIINSG LETVEALAFE
PLSQLLYWVD AGFKKIEVAN PDGDFRLTIV NSSVLDRPRA LVLVPQEGVM FWTDWGDLKP GIYRSNMDGS
AAYHLVSEDV KWPNGISVDD QWIYWTDAYL ECIERITFSG QQRSVILDNL PHPYAIAVFK NEIYWDDWSQ
LSIFRASKYS GSQMEILANQ LTGLMDMKIF YKGKNTGSNA CVPRPCSLLC LPKANNSRSC RCPEDVSSSV
LPSGDLMCDC PQGYQLKNNT CVKEENTCLR NQYRCSNGNC INSIWWCDFD NDCGDMSDER NCPTTICDLD
TQFRCQESGT CIPLSYKCDL EDDCGDNSDE SHCEMHQCRS DEYNCSSGMC IRSSWVCDGD NDCRDWSDEA
NCTAIYHTCE ASNFQCRNGH CIPQRWACDG DTDCQDGSDE DPVNCEKKCN GFRCPNGTCI PSSKHCDGLR
DCSDGSDEQH CEPLCTHFMD FVCKNRQQCL FHSMVCDGII QCRDGSDEDA AFAGCSQDPE FHKVCDEFGF
QCQNGVCISL IWKCDGMDDC GDYSDEANCE NPTEAPNCSR YFQFRCENGH CIPNRWKCDR ENDCGDWSDE**

KDCGDSHILP FSTPGPSTCL PNYYRCSSGT CVMDTWVCDG YRDCADGSDE EACPLLANVT AASTPTQLGR CDRFEFECHQ PKTCIPNWKR CDGHQDCQDG RDEANCPTHS TLTCMSREFQ CEDGEACIVL SERCDGFLDC SDESDEKACS DELTVYKVQN LQWTADFSGD VTLTWMRPKK MPSASCVYNV YYRVVGESIW KTLETHSNKT NTVLKVLKPD TTYQVKVQVQ CLSKAHNTND FVTLRTPEGL PDAPRNLQLS LPREAEGVIV GHWAPPIHTH GLIREYIVEY SRSGSKMWAS QRAASNFTEI KNLLVNTLYT VRVAAVTSRG IGNWSDSKSI TTIKGKVIPP PDIHIDSYGE NYLSFTLTME SDIKVNGYVV NLFWAFDTHK QERRTLNFRG SILSHKVGNL TAHTSYEISA WAKTDLGDSP LAFEHVMTRG VRPPAPSLKA KAINQTAVEC TWTGPRNVVY GIFYATSFLD LYRNPKSLTT SLHNKTVIVS KDEQYLFLVR VVVPYQGPSS DYVVVKMIPD SRLPPRHLHV VHTGKTSVVI KWESPYDSPD QDLLYAIAVK DLIRKTDRSY KVKSRNSTVE YTLNKLEPGG KYHIIVQLGN MSKDSSIKIT TVSLSAPDAL KIITENDHVL LFWKSLALKE KHFNESRGYE IHMFDSAMNI TAYLGNTTDN FFKISNLKMG HNYTFTVQAR CLFGNQICGE PAILLYDELG SGADASATQA ARSTDVAAVV VPILFLILLS LGVGFAILYT KHRRLQSSFT AFANSHYSSR LGSAIFSSGD DLGEDDEDAP MITGFSDDVP MVIA

>IPI:IPI00217882.3 Homo sapiens (Human) SORTILIN PRECURSOR. [MASS=92068]
MERPWGAADG LSRWPHGLGL LLLLQLLPPS TLSQDRLDAP PPPAAPLPRW SGPIGVSWGL RAAAAGGAFP RGGRWRRSAP GEDEECGRVR DFVAKLANNT HQHVFDDLRG SVSLSWVGDS TGVILVLTTF HVPLVIMTFG QSKLYRSEDY GKNFKDITDL INNTFIRTEF GMAIGPENSG KVVLTAEVSG GSRGGRIFRS SDFAKNFVQT DLPFHPLTQM MYSPQNSDYL LALSTENGLW VSKNFGGKWE EIHKAVCLAK WGSDNTIFFT TYANGSCKAD LGALELWRTS DLGKSFKTIG VKIYSFGLGG RFLFASVMAD KDTTRRIHVS TDQGDTWSMA QLPSVGQEQF YSILAANDDM VFMHVDEPGD TGFGTIFTSD DRGIVYSKSL DRHLYTTTGG ETDFTNVTSL RGVYITSVLS EDNSIQTMIT FDQGGRWTHL RKPENSECDA TAKNKNECSL HIHASYSISQ KLNVPMAPLS EPNAVGIVIA HGSVGDAISV MVPDVYISDD GGYSWTKMLE GPHYYTILDS GGIIVAIEHS SRPINVIKFS TDEGQCWQTY TFTRDPIYFT GLASEPGARS MNISIWGFTE SFLTSQWVSY TIDFKDILER NCEEKDYTIW LAHSTDPEDY EDGCILGYKE QFLRLRKSSV CQNGRDYVVT KQPSICLCSL EDFLCDFGYY RPENDSKCVE QPELKGHDLE FCLYGREEHL TTNGYRKIPG DKCQGGVNPV REVKDLKKKC TSNFLSPEKQ NSKSNSVPII LAIVGLMLVT VVAGVLIVKK YVCGGRFLVH RYSVLQQHAE ANGVDGVDAL DTASHTNKSG YHDDSDEDLL E

Protein	Peptide	Cell Line	PSMs	Ratio(BACE/total)
PVR	AKPQNTAEVQK	HEK	6	0.71
PVR	VLAKPQNTAEVQK	HeLa	3	0.75
PVR	HGESGSMAVFHQTQGPSYSESKRLEFVAAR	HEK	2	0.73

>IPI:IPI00299158.1 Homo sapiens (Human) ISOFORM ALPHA OF POLIOVIRUS RECEPTOR PRECURSOR. [MASS=45303]
MARAMAAAWP LLLVALLVLS WPPPGTGDVV VQAPTQVPGF LGDSVTLPCY LQVPNMEVTH VSQLTWARHG ESGSMAVFHQ TQGPSYSESK RLEFVAARLG AELRNASLRM FGLRVEDEGN YTCLFVTFPQ GSRSVDIWLR VLAKPQNTAE VQKVQLTGEP VPMARCVSTG GRPPAQITWH SDLGGMPNTS QVPGFLSGTV TVTSLWILVP SSQVDGKNVT CKVEHESFEK PQLLTVNLTV YYPPEVSISG YDNNWYLGQN EATLTCDARS NPEPTGYNWS TTMGPLPPFA VAQGAQLLIR PVDKPINTTL ICNVTNALGA RQAELTVQVK EGPPSEHSGI SRNAIIFLVL GILVFLILLG IGIYFYWSKC SREVLWHCHL CPSSTEHASA SANGHVSYSA VSRENSSSQD PQTEGTR

Protein	Peptide	Cell Line	PSMs	Ratio(BACE/total)
PCDH21	LLAVEVNTPEK	HEK	3	0.72
PCDH7	ATVVLNIKDENDNVPSIEIRK	HEK	10	0.93
PCDH7	SVYEADLAENSAPGTPILQLR	HEK	4	0.84
PCDHA11	VVVLDVNDHIPMFTQSVYR	HEK	2	0.90
PCDHA5	VVAVDKDSGQNAWLSYR	HEK	1	0.99
PCDHA8	SGSLITAGR	HEK	1	0.95
PCDHC3	VLEDAPSGTR	HEK	1	0.83
PTC21	VHAVDRDTGSGGSVTYFLQNLHSPFAVDR	HEK	7	0.81

>IPI:IPI00040730.4 Homo sapiens (Human) PROTOCADHERIN 21 PRECURSOR. [MASS=93595]
MRRCRWAALA LGLLRLCLAQ ANFAPHFFDN GVGSTNGNMA LFSLPEDTPV GSHVYTLNGT DPEGDPISYH ISFDPSTRSV FSVDPTFGNI TLVEELDRER EDEIEAIISI SDGLNLVAEK VVILVTDAND EAPRFIQEPY

VALVPEDIPA GSIIFKVHAV DRDTGSGGSV TYFLQNLHSP FAVDRHSGVL RLQAGATLDY ERSRTHYITV
VAKDGGGRLH GADVVFSATT TVTVNVEDVQ DMAPVFVGTP YYGYVYEDTL PGSEVLKVVA MDGDRGKPNR
ILYSLVNGND GAFEINETSG AISITQSPAQ LQREVYELHV QVTEMSPAGS PAAQATVPVT IRIVDLNNHP
PTFYGESGPQ NRFELSMNEH PPQGEILRGL KITVNDSDQG ANAKFNLQLV GPRGIFRVVP QTVLNEAQVT
IIVENSAAID FEKSKVLTFK LLAVEVNTPE KFSSTADVVI QLLDTNDNVP KFDSLYYVAR IPENAPGGSS
VVAVTAVDPD TGPWGEVKYS TYGTGADLFL IHPSTGLIYT QPWASLDAEA TARYNFYVKA EDMEGKYSVA
EVFITLLDVN DHPPQFGKSV QKKTMVLGTP VKIEAIDEDA EEPNNLVDYS ITHAEPANVF DINSHTGEIW
LKNSIRSLDA LHNITPGRDC LWSLEVQAKD RGSPSFSTTA LLKIDITDAE TLSRSPMAAF LIQTKDNPMK
AVGVLAGTMA TVVAITVLIS TATFWRNKKS NKVLPMRRVL RKRPSPAPRT IRIEWLKSKS TKAATKFMLK
EKPPNENCNN NSPESSLLPR APALPPPPSV APSTGAAQWT VPTVSGSLTP QPTQPPPKPK TMGSPVQSTL
ISELKQKFEK KSVHNKAYF

>IPI:IPI00001893.2 Homo sapiens (Human) ISOFORM A OF PROTOCADHERIN-7 PRECURSOR. [MASS=116105]

MLRMRTAGWA RGWCLGCCLL LPLSFSLAAA KQLLRYRLAE EGPADVRIGN VASDLGIVTG SGEVTFSLES
GSEYLKIDNL TGELSTSERR IDREKLPQCQ MIFDENECFL DFEVSVIGPS QSWVDLFEGQ VIVLDINDNT
PTFPSPVLTL TVEENRPVGT LYLLPTATDR DFGRNGIERY ELLQEPGGGG SGGESRRAGA ADSAPYPGGG
GNGASGGGSG GSKRRLDASE GGGGTNPGGR SSVFELQVAD TPDGEKQPQL IVKGALDREQ RDSYELTLRV
RDGGDPPRSS QAILRVLITD VNDNSPRFEK SVYEADLAEN SAPGTPILQL RAADLDVGVN GQIEYVFGAA
TESVRRLLRL DETSGWLSVL HRIDREEVNQ LRFTVMARDR GQPPKTDKAT VVLNIKDEND NVPSIEIRKI
GRIPLKDGVA NVAEDVLVDT PIALVQVSDR DQGENGVVTC TVVGDVPFQL KPASDTEGDQ NKKKYFLHTS
TPLDYEATRE FNVVIVAVDS GSPSLSSKNS LIVKVGDTND NPPMFGQSVV EVYFPENNIP GERVATVLAT
DADSGKNAEI AYSLDSSVMG IFAIDPDSGD ILVNTVLDRE QTDRYEFKVN AKDKGIPVLQ GSTTVIVQVA
DKNDNDPKFM QDVFTFYVKE NLQPNSPVGM VTVMDADKGR NAEMSLYIEE NNNIFSIEND TGTIYSTMSF
DREHQTTYTF RVKAVDGGDP PRSATATVSL FVMDENDNAP TVTLPKNISY TLLPPSSNVR TVVATVLATD
SDDGINADLN YSIVGGNPFK LFEIDPTSGV VSLVGKLTQK HYGLHRLVVQ VNDSGQPSQS TTTVVHVFVN
ESVSNATAID SQIARSLHIP LTQDIAGDPS YEISKQRLSI VIGVVAGIMT VILIILIVVM ARYCRSKNKN
GYEAGKKDHE DFFTPQQHDK SKKPKKDKKN KKSKQPLYSS IVTVEASKPN GQRYDSVNEK LSDSPSMGRY
RSVNGGPGSP DLARHYKSSS PLPTVQLHPQ SPTAGKKHQA VQDLPPANTF VGAGDNISIG SDHCSEYSCQ
TNNKYSKQMR LHPYITVFG

>IPI:IPI00012549.1 Homo sapiens (Human) ISOFORM 2 OF PROTOCADHERIN GAMMA A11 PRECURSOR. [MASS=91608]

MANRLQRGDR SRLLLLLCIF LGTLRGFRAR QIRYSVPEET EKGSFVGNIS KDLGLEPREL AKRGVRIVSR
GKTQLFAVNP RSGSLITAGR IDREELCETV SSCFLNMELL VEDTLKIYGV EVEIIDINDN APSFQEDEVE
IKVSEHAIPG ARFALPNARD PDVGVNSLQS YQLSPNNYFS LQLRGRTDGA KNPELVLEGS LDREKEAAHL
LLLTALDGGD PIRKGAVPIR VVVLDVNDHI PMFTQSVYRV SVPENISSGT RVLMVNATDP DEGINGEVMY
SFRNMESKAS EIFQLDSQTG EVQVRGSLDF EKYRFYEMEI QGQDGGGLFT TTTMLITVVD VNDNAPEITI
TSSINSILEN SPPGTVIALL NVQDQDSGEN GQVSCFIPNH LPFKLEKTYG NYYKLITSRV LDRELVQSYN
ITLTATDQGS PPLSAETHVW LNVADDNDNP PVFPHSSYSA YIPENNPRGA SIFSVTALDP DSKQNALVTY
SLTDDTVQGV PLSSYVSINS NTGVLYALQS FDYEQFRDLE LRVIARDSGD PPLSSNVSLS LFVLDQNDNA
PEILYPALPT DGSTGVELAP RSAEPGYLVT KVVAVDKDSG QNAWLSYRLL KASEPGLFAV GEHTGEVRTA
RALLDRDALK QSLVVAVQDH GQPPLSATVT LTVAVADSIP EVLADLGSLE SLANSETSDL SLYLVVAVAA
VSCIFLVFVI VLLALRLWRW HKSRLLQASE GGLAGMPTSH FVGVDGVQAF LQTYSHEVSL IADSQKSHLI
FPQPNYGDTL ISQESCEKSE PLLIAEDSAI ILGKCDPTSN QVRFISLPPN CWCLGTSLLR RCFLSLL

>IPI:IPI00003885.1 Homo sapiens (Human) ISOFORM 1 OF PROTOCADHERIN GAMMA A5 PRECURSOR. [MASS=100935]

MASPPRGWGC GELLLPFMLL GTLCEPGSGQ IRYSMPEELD KGSFVGNIAK DLGLEPQELA ERGVRIVSRG
RTQLFALNPR SGSLVTAGRI DREELCAQSP LCVVNFNILV ENKMKIYGVE VEIIDINDNF PRFRDEELKV
KVNENAAAGT RLVLPFARDA DVGVNSLRSY QLSSNLHFSL DVVSGTDGQK YPELVLEQPL DREKETVHDL
LLTALDGGDP VLSGTTHIRV TVLDANDNAP LFTPSEYSVS VPENIPVGTR LLMLTATDPD EGINGKLTYS
FRNEEEKISE TFQLDSNLGE ISTLQSLDYE ESRFYLMEVV AQDGGALVAS AKVVVTVQDV NDNAPEVILT
SLTSSISEDC LPGTVIALFS VHDGDSGENG EIACSIPRNL PFKLEKSVDN YYHLLTTRDL DREETSDYNI
TLTVMDHGTP PLSTESHIPL KVADVNDNPP NFPQASYSTS VTENNPRGVS IFSVTAHDPD SGDNARVTYS
LAEDTFQGAP LSSYVSINSD TGVLYALRSF DYEQLRDLQL WVTASDSGNP PLSSNVSLSL FVLDQNDNTP
EILYPALPTD GSTGVELAPR SAEPGYLVTK VVAVDKDSGQ NAWLSYRLLK ASEPGLFAVG LHTGEVRTAR

ALLDRDALKQ SLVVAVEDHG QPPLSATFTV TVAVADRIPD ILADLGSIKT PIDPEDLDLT LYLVVAVAAV
SCVFLAFVIV LLVLRLRRWH KSRLLQAEGS RLAGVPASHF VGVDGVRAFL QTYSHEVSLT ADSRKSHLIF
PQPNYADTLL SEESCEKSEP LLMSDKVDAN KEERRVQQAP PNTDWRFSQA QRPGTSGSQN GDDTGTWPNN
QFDTEMLQAM ILASASEAAD GSSTLGGGAG TMGLSARYGP QFTLQHVPDY RQNVYIPGSN ATLTNAAGKR
DGKAPAGGNG NKKKSGKKEK K

>IPI:IPI00012543.1 Homo sapiens (Human) ISOFORM 1 OF PROTOCADHERIN GAMMA A8 PRECURSOR. [MASS=101480]

MAAPQSRPRR GELILLCALL GTLWEIGRGQ IRYSVPEETD KGSFVGNISK DLGLDPRKLA KHGVRIVSRG
RTQLFALNPR SGSLITAGRI DREELCAQSP RCLININTLV EDKGKLFGVE IEIIDINDNN PKFQVEDLEV
KINEIAVPGA RYPLPEAVDP DVGVNSLQSY QLSPNHHFSL DVQTGDNGAI NPELVLERAL DREEEAAHHL
VLTASDGGKP PRSSTVRIHV TVLDTNDNAP VFPHPIYRVK VLENMPPGTR LLTVTASDPD EGINGKVAYK
FRKINEKQTP LFQLNENTGE ISIAKSLDYE ECSFYEMEIQ AEDVGALLGR TKLLISVEDV NDNRPEVIIT
SLFSPVLENS LPGTVIAFLS VHDQDSGKNG QVVCYTRDNL PFKLEKSIGN YYRLVTRKYL DRENVSIYNI
TVMASDLGTP PLSTETQIAL HVADINDNPP TFPHASYSAY ILENNLRGAS IFSLTAHDPD SQENAQVTYS
VTEDTLQGAP LSSYISINSD TGVLYALQSF DYEQIRDLQL LVTASDSGDP PLSSNMSLSL FVLDQNDNAP
EILYPALPTD GSTGVELAPR SAERGYLVTK VVAVDRDSGQ NAWLSYRLLK ASEPGLFSVG LHTGEVRTAR
ALLDRDALKQ SLVVAVQDHG QPPLSATVTL TVAVADSIPE VLTELGSLKP SVDPNDSSLT LYLVVAVAAI
SCVFLAFVAV LLGLRLRRWH KSRLLQDSGG RLVGVPASHF VGVEEVQAFL QTYSQEVSLT ADSRKSHLIF
PQPNYADMLI SQEGCEKNDS LLTSVDFHEY KNEADHGQQA PPNTDWRFSQ AQRPGTSGSQ NGDDTGTWPN
NQFDTEMLQA MILASASEAA DGSSTLGGGA GTMGLSARYG PQFTLQHVPD YRQNVYIPGS NATLTNAAGK
RDGKAPAGGN GNKKKSGKKE KK

>IPI:IPI00001872.3 Homo sapiens (Human) ISOFORM 1 OF PROTOCADHERIN GAMMA C3 PRECURSOR. [MASS=101077]

MVPEAWRSGL VSTGRVVGVL LLLGALNKAS TVIHYEIPEE REKGFAVGNV VANLGLDLGS LSARRFRVVS
GASRRFFEVN RETGEMFVND RLDREELCGT LPSCTVTLEL VVENPLELFS VEVVIQDIND NNPAFPTQEM
KLEISEAVAP GTRFPLESAH DPDVGSNSLQ TYELSRNEYF ALRVQTREDS TKYAELVLER ALDREREPSL
QLVLTALDGG TPALSASLPI HIKVLDANDN APVFNQSLYR ARVLEDAPSG TRVVQVLATD LDEGPNGEII
YSFGSHNRAG VRQLFALDLV TGMLTIKGRL DFEDTKLHEI YIQAKDKGAN PEGAHCKVLV EVVDVNDNAP
EITVTSVYSP VPEDAPLGTV IALLSVTDLD AGENGLVTCE VPPGLPFSLT SSLKNYFTLK TSADLDRETV
PEYNLSITAR DAGTPSLSAL TIVRVQVSDI NDNPPQSSQS SYDVYIEENN LPGAPILNLS VWDPDAPQNA
RLSFFLLEQG AETGLVGRYF TINRDNGIVS SLVPLDYEDR REFELTAHIS DGGTPVLATN ISVNIFVTDR
NDNAPQVLYP RPGGSSVEML PRGTSAGHLV SRVVGWDADA GHNAWLSYSL LGSPNQSLFA IGLHTGQIST
ARPVQDTDSP RQTLTVLIKD NGEPSLSTTA TLTVSVTEDS PEARAEFPSG SAPREQKKNL TFYLLLSLIL
VSVGFVVTVF GVIIFKVYKW KQSRDLYRAP VSSLYRTPGP SLHADAVRGG LMSPHLYHQV YLTTDSRRSD
PLLKKPGAAS PLASRQNTLR SCDPVFYRQV LGAESAPPGQ QAPPNTDWRF SQAQRPGTSG SQNGDDTGTW
PNNQFDTEML QAMILASASE AADGSSTLGG GAGTMGLSAR YGPQFTLQHV PDYRQNVYIP GSNATLTNAA
GKRDGKAPAG GNGNKKKSGK KEKK

>IPI:IPI00040730.4 Homo sapiens (Human) PROTOCADHERIN 21 PRECURSOR. [MASS=93595]

MRRCRWAALA LGLLRLCLAQ ANFAPHFFDN GVGSTNGNMA LFSLPEDTPV GSHVYTLNGT DPEGDPISYH
ISFDPSTRSV FSVDPTFGNI TLVEELDRER EDEIEAIISI SDGLNLVAEK VVILVTDAND EAPRFIQEPY
VALVPEDIPA GSIIFKVHAV DRDTGSGGSV TYFLQNLHSP FAVDRHSGVL RLQAGATLDY ERSRTHYITV
VAKDGGGRLH GADVVFSATT TVTVNVEDVQ DMAPVFVGTP YYGYVYEDTL PGSEVLKVVA MDGDRGKPNR
ILYSLVNGND GAFEINETSG AISITQSPAQ LQREVYELHV QVTEMSPAGS PAAQATVPVT IRIVDLNNHP
PTFYGESGPQ NRFELSMNEH PPQGEILRGL KITVNDSDQG ANAKFNLQLV GPRGIFRVVP QTVLNEAQVT
IIVENSAAID FEKSKVLTFK LLAVEVNTPE KFSSTADVVI QLLDTNDNVP KFDSLYYVAR IPENAPGGSS
VVAVTAVDPD TGPWGEVKYS TYGTGADLFL IHPSTGLIYT QPWASLDAEA TARYNFYVKA EDMEGKYSVA
EVFITLLDVN DHPPQFGKSV QKKTMVLGTP VKIEAIDEDA EEPNNLVDYS ITHAEPANVF DINSHTGEIW
LKNSIRSLDA LHNITPGRDC LWSLEVQAKD RGSPSFSTTA LLKIDITDAE TLSRSPMAAF LIQTKDNPMK
AVGVLAGTMA TVVAITVLIS TATFWRNKKS NKVLPMRRVL RKRPSPAPRT IRIEWLKSKS TKAATKFMLK
EKPPNENCNN NSPESSLLPR APALPPPPSV APSTGAAQWT VPTVSGSLTP QPTQPPPKPK TMGSPVQSTL
ISELKQKFEK KSVHNKAYF

Protein	Peptide	Cell Line	PSMs	Ratio(BACE/total)
PXDC2	EITVATGGFIYTGEVVHR	HEK	1	0.81
PXDC2	EIPVLVTQISSTNHPVK	HeLa	5	0.77
PXDC2	HRQDWVDSGCPEESKEK	HEK	4	0.85

>IPI:IPI00044369.2 Homo sapiens (Human) ISOFORM 1 OF PLEXIN DOMAIN-CONTAINING PROTEIN 2 PRECURSOR. [MASS=59583]

**MARFPKADLA AAGVMLLCHF FTDQFQFADG KPGDQILDWQ YGVTQAFPHT EEEVEVDSHA YSHRWKRNLD
FLKAVDTNRA SVGQDSPEPR SFTDLLLDDG QDNNTQIEED TDHNYYISRI YGPSDSASRD LWVNIDQMEK
DKVKIHGILS NTHRQAARVN LSFDFPFYGH FLREITVATG GFIYTGEVVH RMLTATQYIA PLMANFDPSV
SRNSTVRYFD NGTALVVQWD HVHLQDNYNL GSFTFQATLL MDGRIIFGYK EIPVLVTQIS STNHPVKVGL
SDAFVVVHRI QQIPNVRRRT IYEYHRVELQ MSKITNISAV EMTPLPTCLQ FNRCGPCVSS QIGFNCSWCS
KLQRCSSGFD RHRQDWVDSG CPEESKEKMC ENTEPVETSS RTTTTVGATT TQFRVLTTTR RAVTSQFPTS
LPTEDDTKIA LHLKDNGAST DDSAAEKKGG TLHAGLIIGI LILVLIVATA ILVTVYMYHH PTSAASIFFI
ERRPSRWPAM KFRRGSGHPA YAEVEPVGEK EGFIVSEQC**

Protein	Peptide	Cell Line	PSMs	Ratio(BACE/total)
DSG2	GNNVEKPLELR	HEK	1	0.95

>IPI:IPI00028931.2 Homo sapiens (Human) DESMOGLEIN 2. [MASS=122294]

**MARSPGRAYA LLLLLICFNV GSGLHLQVLS TRNENKLLPK HPHLVRQKRA WITAPVALRE GEDLSKKNPI
AKIHSDLAEE RGLKITYKYT GKGITEPPFG IFVFNKDTGE LNVTSILDRE ETPFFLLTGY ALDARGNNVE
KPLELRIKVL DINDNEPVFT QDVFVGSVEE LSAAHTLVMK INATDADEPN TLNSKISYRI VSLEPAYPPV
FYLNKDTGEI YTTSVTLDRE EHSSYTLTVE ARDGNGEVTD KPVKQAQVQI RILDVNDNIP VVENKVLEGM
VEENQVNVEV TRIKVFDADE IGSDNWLANF TFASGNEGGY FHIETDAQTN EGIVTLIKEV DYEEMKNLDF
SVIVANKAAF HKSIRSKYKP TPIPIKVKVK NVKEGIHFKS SVISIYVSES MDRSSKGQII GNFQAFDEDT
GLPAHARYVK LEDRDNWISV DSVTSEIKLA KLPDFESRYV QNGTYTVKIV AISEDYPRKT ITGTVLINVE
DINDNCPTLI EPVQTICHDA EYVNVTAEDL DGHPNSGPFS FSVIDKPPGM AEKWKIARQE STSVLLQQSE
KKLGRSEIQF LISDNQGFSC PEKQVLTLTV CECLHGSGCR EAQHDSYVGL GPAAIALMIL AFLLLLLVPL
LLLMCHCGKG AKGFTPIPGT IEMLHPWNNE GAPPEDKVVP SFLPVDQGGS LVGRNGVGGM AKEATMKGSS
SASIVKGQHE MSEMDGRWEE HRSLLSGRAT QFTGATGAIM TTETTKTARA TGASRDMAGA QAAAVALNEE
FLRNYFTDKA ASYTEEDENH TAKDCLLVYS QEETESLNAS IGCCSFIEGE LDDRFLDDLG LKFKTLAEVC
LGQKIDINKE IEQRQKPATE TSMNTASHSL CEQTMVNSEN TYSSGSSFPV PKSLQEANAE KVTQEIVTER
SVSSRQAQKV ATPLPDPMAS RNVIATETSY VTGSTMPPTT VILGPSQPQS LIVTERVYAP ASTLVDQPYA
NEGTVVVTER VIQPHGGGSN PLEGTQHLQD VPYVMVRERE SFLAPSSGVQ PTLAMPNIAV GQNVTVTERV
LAPASTLQSS YQIPTENSMT ARNTTVSGAG VPGPLPDFGL EESGHSNSTI TTSSTRVTKH STVQHSYS**

Protein	Peptide	Cell Line	PSMs	Ratio(BACE/total)
BACE1	ETDEEPEEPGRR	HEK	5	0.89
BACE1	SIVDSGTTNLRLPK	HEK	3	0.91
BACE1	GSFVEMVDNLR	HEK	1	0.93
BACE1	SIVDSGTTNLR	HEK	1	0.87

>IPI:IPI00011518.1 Homo sapiens (Human) ISOFORM A OF BETA-SECRETASE 1 PRECURSOR. [MASS=55764]

**MAQALPWLLL WMGAGVLPAH GTQHGIRLPL RSGLGGAPLG LRLPRETDEE PEEPGRRGSF VEMVDNLRGK
SGQGYYVEMT VGSPPQTLNI LVDTGSSNFA VGAAPHPFLH RYYQRQLSST YRDLRKGVYV PYTQGKWEGE
LGTDLVSIPH GPNVTVRANI AAITESDKFF INGSNWEGIL GLAYAEIARP DDSLEPFFDS LVKQTHVPNL
FSLQLCGAGF PLNQSEVLAS VGGSMIIGGI DHSLYTGSLW YTPIRREWYY EVIIVRVEIN GQDLKMDCKE
YNYDKSIVDS GTTNLRLPKK VFEAAVKSIK AASSTEKFPD GFWLGEQLVC WQAGTTPWNI FPVISLYLMG
EVTNQSFRIT ILPQQYLRPV EDVATSQDDC YKFAISQSST GTVMGAVIME GFYVVFDRAR KRIGFAVSAC
HVHDEFRTAA VEGPFVTLDM EDCGYNIPQT DESTLMTIAY VMAAICALFM LPLCLMVCQW RCLRCLRQQH
DDFADDISLL K**

Protein	Peptide	Cell Line	PSMs	Ratio(BACE/total)
ITFG1	SANFLDHLYVGIPR	HEK	5	0.97

>IPI:IPI00006547.4 Homo sapiens (Human) T-CELL IMMUNOMODULATORY PROTEIN PRECURSOR. [MASS=68108]

**MAAAGRLPSS WALFSPLLAG LALLGVGPVP ARALHNVTAE LFGAEAWGTL AAFGDLNSDK QTDLFVLRER
NDLIVFLADQ NAPYFKPKVK VSFKNHSALI TSVVPGDYDG DSQMDVLLTY LPKNYAKSEL GAVIFWGQNQ
TLDPNNMTIL NRTFQDEPLI MDFNGDLIPD IFGITNESNQ PQILLGGNLS WHPALTTTSK MRIPHSHAFI
DLTEDFTADL FLTTLNATTS TFQFEIWENL DGNFSVSTIL EKPQNMMVVG QSAFADFDGD GHMDHLLPGC
EDKNCQKSTI YLVRSGMKQW VPVLQDFSNK GTLWGFVPFV DEQQPTEIPI PITLHIGDYN MDGYPDALVI
LKNTSGSNQQ AFLLENVPCN NASCEEARRM FKVYWELTDL NQIKDAMVAT FFDIYEDGIL DIVVLSKGYT
KNDFAIHTLK NNFEADAYFV KVIVLSGLCS NDCPRKITPF GVNQPGPYIM YTTVDANGYL KNGSAGQLSQ
SAHLALQLPY NVLGLGRSAN FLDHLYVGIP RPSGEKSIRK QEWTAIIPNS QLIVIPYPHN VPRSWSAKLY
LTPSNIVLLT AIALIGVCVF ILAIIGILHW QEKKADDREK RQEAHRFHFD AM**

Protein	Peptide	Cell Line	PSMs	Ratio(BACE/total)
UNC5C	NEDIIDPVEDRNFYITIDHNLIIK	HEK	8	0.92

>IPI:IPI00293757.3 Homo sapiens (Human) ISOFORM 1 OF NETRIN RECEPTOR UNC5C PRECURSOR. [MASS=103102]

**MRKGLRATAA RCGLGLGYLL QMLVLPALAL LSASGTGSAA QDDDFFHELP ETFPSDPPEP LPHFLIEPEE
AYIVKNKPVN LYCKASPATQ IYFKCNSEWV HQKDHIVDER VDETSGLIVR EVSIEISRQQ VEELFGPEDY
WCQCVAWSSA GTTKSRKAYV RIAYLRKTFE QEPLGKEVSL EQEVLLQCRP PEGIPVAEVE WLKNEDIIDP
VEDRNFYITI DHNLIIKQAR LSDTANYTCV AKNIVAKRKS TTATVIVYVN GGWSTWTEWS VCNSRCGRGY
QKRTRTCTNP APLNGGAFCE GQSVQKIACT TLCPVDGRWT PWSKWSTCGT ECTHWRRREC TAPAPKNGGK
DCDGLVLQSK NCTDGLCMQT APDSDDVALY VGIVIAVIVC LAISVVVALF VYRKNHRDFE SDIIDSSALN
GGFQPVNIKA ARQDLLAVPP DLTSAAAMYR GPVYALHDVS DKIPMTNSPI LDPLPNLKIK VYNTSGAVSP
QDDLSEFTSK LSPQMTQSLL ENEALSLKNQ SLARQTDPSC TAFGSFNSLG GHLIVPNSGV SLLIPAGAIP
QGRVYEMYVT VHRKETMRPP MDDSQTLLTP VVSCGPPGAL LTRPVVLTMH HCADPNTEDW KILLKNQAAQ
GQWEDVVVVG EENFTTPCYI KLDAEACHIL TENLSTYALV GHSTTKAAAK RLKLAIFGPL CCSSLEYSIR
VYCLDDTQDA LKEILHLERQ TGGQLLEEPK ALHFKGSTHN LRLSIHDIAH SLWKSKLLAK YQEIPFYHVW
SGSQRNLHCT FTLERFSLNT VELVCKLCVR QVEGEGQIFQ LNCTVSEEPT GIDLPLLDPA NTITTVTGPS
AFSIPLPIRQ KLCSSLDAPQ TRGHDWRMLA HKLNLDRYLN YFATKSSPTG VILDLWEAQN FPDGNLSMLA
AVLEEMGRHE TVVSLAAEGQ Y**

<u>GPI and Type II Transmembrane Proteins</u>

Protein	Peptide	Cell Line	PSMs	Ratio(BACE/total)
CNTN1	VIIECKPK	HEK	3	0.88
CNTN1	VTVTNPDTGR	HEK	1	0.86

GPI Anchor

>IPI:IPI00029751.1 Homo sapiens (Human) ISOFORM 1 OF CONTACTIN-1 PRECURSOR. [MASS=113320]

**MKMWLLVSHL VIISITTCLA EFTWYRRYGH GVSEEDKGFG PIFEEQPINT IYPEESLEGK VSLNCRARAS
PFPVYKWRMN NGDVDLTSDR YSMVGGNLVI NNPDKQKDAG IYYCLASNNY GMVRSTEATL SFGYLDPFPP
EERPEVRVKE GKGMVLLCDP PYHFPDDLSY RWLLNEFPVF ITMDKRRFVS QTNGNLYIAN VEASDKGNYS
CFVSSPSITK SVFSKFIPLI PIPERTTKPY PADIVVQFKD VYALMGQNVT LECFALGNPV PDIRWRKVLE
PMPSTAEIST SGAVLKIFNI QLEDEGIYEC EAENIRGKDK HQARIYVQAF PEWVEHINDT EVDIGSDLYW
PCVATGKPIP TIRWLKNGYA YHKGELRLYD VTFENAGMYQ CIAENTYGAI YANAELKILA LAPTFEMNPM
KKKILAAKGG RVIIECKPKA APKPKFSWSK GTEWLVNSSR ILIWEDGSLE INNITRNDGG IYTCFAENNR
GKANSTGTLV ITDPTRIILA PINADITVGE NATMQCAASF DPALDLTFVW SFNGYVIDFN KENIHYQRNF
MLDSNGELLI RNAQLKHAGR YTCTAQTIVD NSSASADLVV RGPPGPPGGL RIEDIRATSV ALTWSRGSDN
HSPISKYTIQ TKTILSDDWK DAKTDPPIIE GNMEAARAVD LIPWMEYEFR VVATNTLGRG EPSIPSNRIK
TDGAAPNVAP SDVGGGGGRN RELTITWAPL SREYHYGNNF GYIVAFKPFD GEEWKKVTVT NPDTGRYVHK
DETMSPSTAF QVKVKAFNNK GDGPYSLVAV INSAQDAPSE APTEVGVKVL SSSEISVHWE HVLEKIVESY
QIRYWAAHDK EEAANRVQVT SQEYSARLEN LLPDTQYFIE VGACNSAGCG PPSDMIEAFT KKAPPSQPPR
IISSVRSGSR YIITWDHVVA LSNESTVTGY KVLYRPDGQH DGKLYSTHKH SIEVPIPRDG EYVVEVRAHS
DGGDGVVSQV KISGAPTLSP SLLGLLLPAF GILVYLEF**

Protein	Peptide	Cell Line	PSMs	Ratio(BACE/total)
EFNA5	VFVRPTNSCMK	HEK	1	0.74
EFNA5	YVLYMVNFDGYSACDHTSK	HeLa	1	0.68

GPI Anchor

>IPI:IPI00005517.1 Homo sapiens (Human) EPHRIN-A5 PRECURSOR. [MASS=26297]

**MLHVEMLTLV FLVLWMCVFS QDPGSKAVAD RYAVYWNSSN PRFQRGDYHI DVCINDYLDV FCPHYEDSVP
EDKTERYVLY MVNFDGYSAC DHTSKGFKRW ECNRPHSPNG PLKFSEKFQL FTPFSLGFEF RPGREYFYIS
SAIPDNGRRS CLKLKVFVRP TNSCMKTIGV HDRVFDVNDK VENSLEPADD TVHESAEPSR GENAAQTPRI
PSRLLAILLF LLAMLLTL**

Protein	Peptide	Cell Line	PSMs	Ratio(BACE/total)
GPC3	VAHVEHEETLSSR	HEK	1	0.78
GPC3	VAHVEHEETLSSR	HEK	1	0.71

GPI Anchor

>IPI:IPI00019907.1 Homo sapiens (Human) GLYPICAN-3 PRECURSOR. [MASS=65563]

**MAGTVRTACL VVAMLLSLDF PGQAQPPPPP PDATCHQVRS FFQRLQPGLK WVPETPVPGS DLQVCLPKGP
TCCSRKMEEK YQLTARLNME QLLQSASMEL KFLIIQNAAV FQEAFEIVVR HAKNYTNAMF KNNYPSLTPQ
AFEFVGEFFT DVSLYILGSD INVDDMVNEL FDSLFPVIYT QLMNPGLPDS ALDINECLRG ARRDLKVFGN
FPKLIMTQVS KSLQVTRIFL QALNLGIEVI NTTDHLKFSK DCGRMLTRMW YCSYCQGLMM VKPCGGYCNV
VMQGCMAGVV EIDKYWREYI LSLEELVNGM YRIYDMENVL LGLFSTIHDS IQYVQKNAGK LTTTIGKLCA
HSQQRQYRSA YYPEDLFIDK KVLKVAHVEH EETLSSRRRE LIQKLKSFIS FYSALPGYIC SHSPVAENDT
LCWNGQELVE RYSQKAARNG MKNQFNLHEL KMKGPEPVVS QIIDKLKHIN QLLRTMSMPK GRVLDKNLDE
EGFESGDCGD DEDECIGGSG DGMIKVKNQL RFLAELAYDL DVDDAPGNSQ QATPKDNEIS TFHNLGNVHS
PLKLLTSMAI SVVCFFFLVH**

Protein	Peptide	Cell Line	PSMs	Ratio(BACE/total)
GOLIM4	LAVQQVEEAQQLREHQEALHQQR	HEK	4	0.85
GOLIM4	LAVQQVEEAQQLR	HEK	1	0.99

Type II Transmembrane -

>IPI:IPI00004962.1 Homo sapiens (Human) GOLGI PHOSPHOPROTEIN 4. [MASS=81880]

MGNGMCSRKQ KRIFQTLLLL TVVFGFLYGA MLYYELQTQL RKAEAVALKY QQHQESLSAQ LQVVYEHRSR
LEKSLQKERL EHKKAKEDFL VYKLEAQETL NKGRQDSNSR YSALNVQHQM LKSQHEELKK QHSDLEEEHR
KQGEDFSRTF NDHKQKYLQL QQEKEQELSK LKETVYNLRE ENRQLRKAHQ DIHTQLQDVK QQHKNLLSEH
EQLVVTLEDH KSALAAAQTQ VAEYKQLKDT LNRIPSLRKP DPAEQQNVTQ VAHSPQGYNT AREKPTREVQ
EVSRNNDVWQ NHEAVPGRAE DTKLYAPTHK EAEFQAPPEP IQQEVERREP EEHQVEEEHR KALEEEEMEQ
VGQAEHLEEE HDPSPEEQDR EWKEQHEQRE AANLLEGHAR AEVYPSAKPM IKFQSPYEEQ LEQQRLAVQQ
VEEAQQLREH QEALHQQRLQ GHLLRQQEQQ QQQVAREMAL QRQAELEEGR PQHQEQLRQQ AHYDAMDNDI
VQGAEDQGIQ GEEGAYERDN QHQDEAEGDP GNRHEPREQG PREADPESEA DRAAVEDINP ADDPNNQGED
EFEEAEQVRE ENLPDENEEQ KQSNQKQENT EVEEHLVMAG NPDQQEDNVD EQYQEEAEEE VQEDLTEEKK
RELEHNAEET YGENDENTDD KNNDGEEQEV RDDNRPKGRE EHYEEEEEEE EDGAAVAEKS HRRAEM
